# NIR Luminescence from Deep-Level Traps in CsPbBr_3_ Microcrystals

**DOI:** 10.1021/acs.jpclett.5c00545

**Published:** 2025-03-31

**Authors:** Jonathan Vandenwijngaerden, Bapi Pradhan, Bob Van Hout, Eduard Fron, Yasuyuki Araki, Xianjun Zhang, Yutaka Shibata, Dario Santantonio, Roger Bresoli-Obach, Santi Nonell, Haifeng Yuan, Jialiang Xu, Mark Van der Auweraer, Maarten Roeffaers, Johan Hofkens, Hiroshi Fukumura, Elke Debroye

**Affiliations:** † Molecular Imaging and Photonics, Department of Chemistry, KU Leuven, Celestijnenlaan 200F, 3001 Leuven, Belgium; ‡ Institute of Multidisciplinary Research for Advanced Materials, Tohoku University, Katahira 2-1-1, Aoba-ku, Sendai, Japan 980-8577; § Department of Chemistry, Graduate School of Science, Tohoku University, 6-3 Aramaki Aza-Aoba, Aoba-ku, Sendai, Japan 980-0578; ∥ Department of Chemistry, Massachusetts Institute of Technology, Cambridge, Massachusetts 02139, United States; ⊥ AppLightChem, Institut Químic de Sarrià, Universitat Ramon Llull, Via Augusta 390, Barcelona, Catalunya 08017, Spain; # Yongjiang Laboratory, 1792 Cihai South Road, Ningbo 315202, China; 7 School of Materials Science and Engineering, Tianjin Key Laboratory of Metal and Molecular Materials Chemistry, Frontiers Science Center for New Organic Matter, Nankai University, Tongyan Road 38, Tianjin 300350, China; 8 cMACS, Department of Microbial and Molecular Systems, KU Leuven, Celestijnenlaan 200F, 3001 Leuven, Belgium; 9 Max Planck Institute for Polymer Research, Ackermannweg 10, 55128 Mainz, Germany

## Abstract

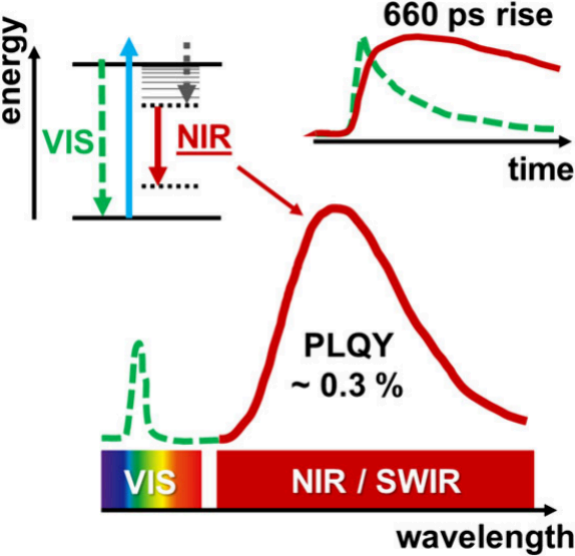

In this study, we report the first observation of a near-infrared (NIR) emission band from all-inorganic CsPbBr_3_ and CsPb(Br/Cl)_3_ perovskite microcrystals. By means of temperature- and power-dependent NIR and visible luminescence spectroscopy, we demonstrate that a fraction of the excited states in these materials relax through radiative transitions involving traps located deep within the band gap, leading to broadband NIR emission. The quantum yield of this deep trap emission is quantitatively determined for the first time and amounts to approximately 0.3% at room temperature. Furthermore, by examining the picosecond-to-nanosecond dynamics of the excited states, using time-resolved luminescence spectroscopy, we observe that the population of NIR initial states occurs on a 660 ps time scale, consistent with the capture of free carriers by deep trap sites. Hence, this work deepens our fundamental understanding of previously unexplored recombination channels in metal halide perovskite microcrystals.

Metal halide perovskites (MHPs) such as CsPbBr_3_ have become immensely attractive semiconductor materials in past years, with exciting applications in photovoltaics,^1,2^ light-emitting diodes,^3−5^ photodetectors,^6,7^ and photocatalysis.^8−12^ Their popularity results from the combination of their easy, low-cost, solution-based processability with favorable optoelectronic properties, including high absorption coefficients,^13^ long carrier diffusion lengths,^14^ and tunable band gaps.^15,16^ Recently, to upscale the production of MHPs for optoelectronic devices such as large-area X-ray detectors,^7^ MHP microcrystals are gaining interest. Compared to MHP nanocrystals, the excited-state relaxation in microcrystals is far less affected by surface defects and charge separation is more efficient as it is not hindered by quantum confinement.^17−20^ While MHP nanocrystals are characterized by a well-defined emission band with a near-unity photoluminescence quantum yield (PLQY) in colloidal solution,^21,22^ larger MHP materials usually have more complex emission spectra and very low PLQYs,^23^ indicating that several radiative and nonradiative channels dominate the relaxation process. To further improve the performance of microcrystalline MHPs, a fundamental understanding of their photophysical processes and excited state decay channels is necessary. Identifying the decay channels is required in order to prevent unwanted recombination when efficient charge separation or the emission of visible light is desired, for instance, in solar cells and visible-range LEDs. In this context, Motti et al.^24^ discovered that intrinsic point defects in polycrystalline MAPbBr_3_ and MAPbI_3_ films, such as halide interstitials and lead vacancies, create deep levels within the band gap where charge carriers can be trapped and subsequently recombine radiatively, generating near-infrared (NIR) luminescence. It was, however, difficult to assess the relative contribution of this decay channel, owing to the lack of a quantitative value for the NIR PLQY. Furthermore, Motti et al. did not determine an activation energy for the thermal quenching of the NIR emission based on temperature-dependent NIR spectra, while this can give valuable information about the position of the energy levels involved in the NIR emission. From an application viewpoint, these recombination channels involving deep trap sites can be utilized to develop new light sources beyond the visible part of the electromagnetic spectrum. For example, the second NIR window (NIR-II) (1000–1400 nm) has emerged as a promising wavelength range for sensitive *in vivo* bioimaging because of the high penetration depth and reduced photodamage to vulnerable samples.^25,26^ All-inorganic perovskite materials have a significant advantage in this respect over hybrid organic–inorganic perovskites due to their high stability under ambient conditions, which is essential for commercial applications.^6,27−30^

In this Letter, we report the first observation of a NIR emission band from all-inorganic CsPbBr_3_ and CsPb(Br/Cl)_3_ perovskite microcrystals at room temperature under ambient conditions. We combine several steady-state and time-resolved spectroscopy techniques to study the origin and excited-state dynamics of the below-band-gap NIR emission. More specifically, we provide an estimation for the NIR PLQY, analyze the effect of temperature, discuss the picosecond–nanosecond excited-state processes, which are responsible for the population and decay of the deep traps yielding NIR emission, and propose an energy-level diagram to interpret the NIR emission of CsPbBr_3_ and CsPb(Br/Cl)_3_ microcrystals.

CsPbBr_3_ and CsPbBr_2.6_Cl_0.4_ (CsPb(Br/Cl)_3_) microcrystals were prepared at room temperature under ambient conditions via a conventional solution-based protocol according to Huang et al.^31^ The Br:Cl ratio of CsPbBr_2.6_Cl_0.4_ obtained using energy-dispersive X-ray spectroscopy (EDX) amounts to a Cl content of about 14%, which is lower than what would be expected from equimolar amounts of Br and Cl in the precursors. This is possibly due to the formation of CsCl, which has poor solubility in DMSO,^32^ and to the use of HBr to precipitate the MCs. Further details regarding the synthesis as well as the structural and morphological features of the microcrystals are provided in the Supporting Information (Figure S1, SI). The electronic band gap of CsPbBr_3_ was found to be 2.38 eV (SI), which is in agreement with the value commonly observed for bulk CsPbBr_3_ perovskite samples.^33−35^ Due to the incorporation of chlorine, the absorption edge of CsPb(Br/Cl)_3_ is at higher energies compared to CsPbBr_3_ (Figure 1A), resulting in an increased band gap energy of 2.45 eV.

**Figure 1:**
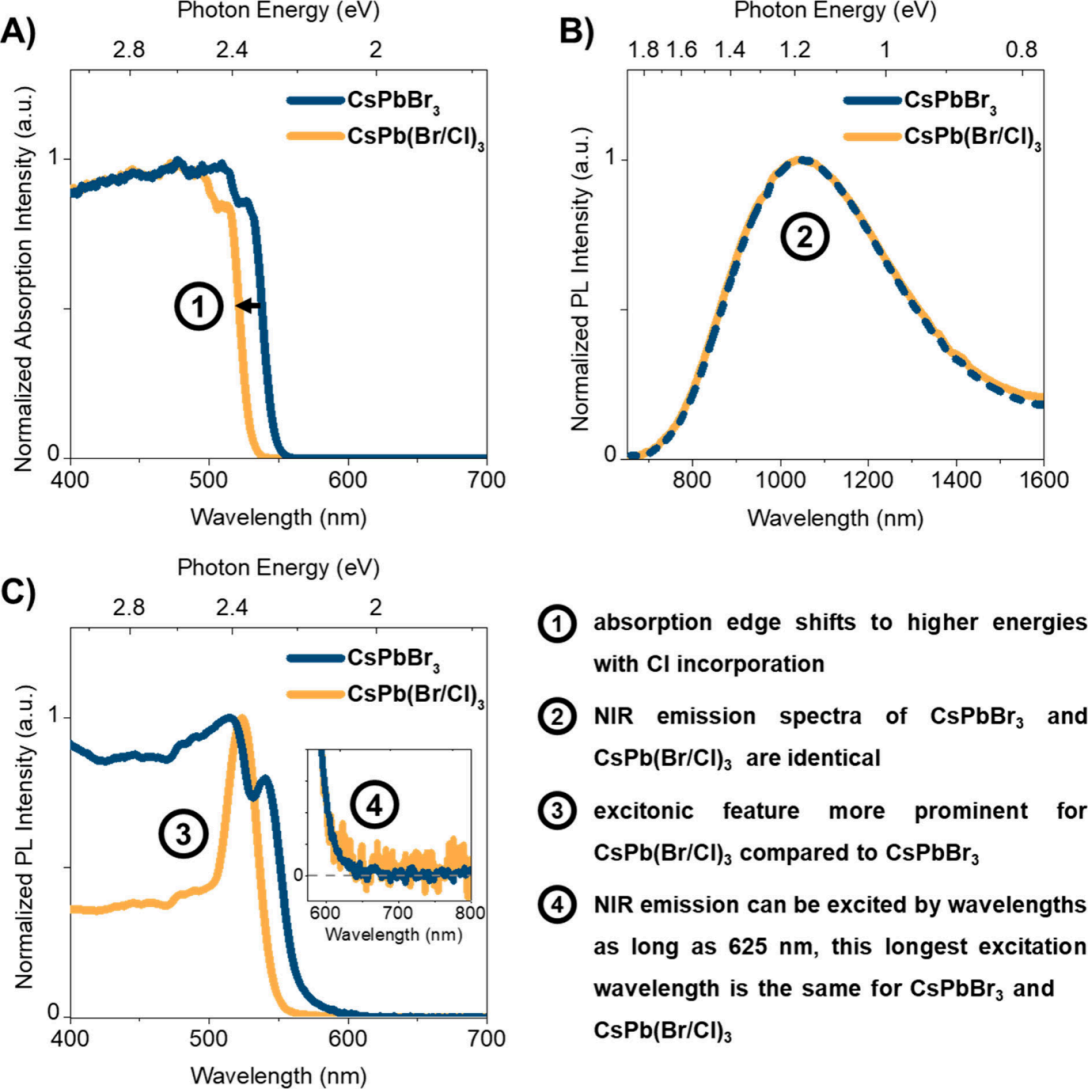
Steady-state spectroscopy at RT. A) Kubelka–Munk equivalent absorption spectra. B) NIR emission spectra (exc. 450 nm). C) NIR excitation spectra (det. 1150 nm) normalized to the maximum (inset: expansion (×100) of NIR excitation spectra (det. 1150 nm), normalized at 570 nm.

Under blue (450 nm) or near-ultraviolet (400 nm) light excitation, the CsPbBr_3_ and CsPb(Br/Cl)_3_ microcrystals generate, by analogy to what was observed for MAPbI_3_, photoluminescence (PL) in the visible (VIS) and the NIR regions.^24^ The visible emission spectrum of CsPbBr_3_ (Figure S5, SI) at room temperature consists of two peaks around 522 nm (2.38 eV), attributed to band-to-band and free exciton recombination, and 546 nm (2.27 eV), usually ascribed to the recombination of excitons bound to (shallow) trap sites,^7,36,37^ with a very low PLQY (∼0.005%). For CsPb(Br/Cl)_3_, the maxima are shifted to 515 nm (2.41 eV) and 533 nm (2.33 eV). This weak, double-peak visible emission has been reported for CsPbBr_3_ microcrystals^7^ and single crystals.^36,37^ More remarkably, the CsPbBr_3_ and CsPb(Br/Cl)_3_ microcrystals show a broad, featureless PL band in the NIR (Figure 1B, Table 1). Using an NIR-sensitive InGaAs camera (detection range from 950 to 1700 nm), direct images of NIR-emitting CsPbBr_3_ microcrystals were readily recorded at room temperature (Figure S2, SI). The NIR emission spectra of CsPbBr_3_ and CsPb(Br/Cl)_3_ share the same maximum at ∼1054 nm (1.18 eV) and have an identical band shape, with a full width at half-maximum (fwhm) of ∼0.49 eV. The position of the emission maximum does not change by altering the excitation wavelength (Figure S4, SI).

**Table 1:** Summary of Steady-State Photophysical Properties

Sample	Band gap energy (eV)	NIR emission maximum (eV)	Energy difference between CBM and lowest energy of NIR excitation spectrum (eV)	NIR activation energy (eV)	VIS activation energy (eV)	NIR QY (%)
CsPbBr_3_	2.38	1.18	0.40	0.36 ± 0.02	0.05 ± 0.02	0.3 ± 0.2 (exc. 450 nm)
CsPb(Br/Cl)_3_	2.45	1.18	0.47	0.42 ± 0.05	0.06 ± 0.01	0.3 ± 0.3 (exc. 450 nm)
						0.7 ± 0.5 (exc. 530 nm)

The NIR PL excitation spectrum of CsPbBr_3_ (Figure 1C), detected at 1150 nm, has a structure which is typical for perovskite absorption and VIS PL excitation spectra, with an excitonic resonance peak (540 nm, 2.30 eV) and an extended excitation edge that continues toward higher energies. The former corresponds to the transition from the ground state to an exciton state, in which an electron and a hole are weakly bound by Coulombic interactions and move together through the lattice, whereas the latter is ascribed to band-to-band excitation that creates free electrons in the conduction band and free holes in the valence band.^38^ Compared to the Kubelka–Munk equivalent absorption spectrum, the rising edge of the excitation spectrum is less steep and extends even to 625 nm (1.98 eV) for CsPbBr_3_ (Figure 1C, inset). This extension to longer wavelengths suggests that the NIR emission also originates from direct excitation of sub-band-gap states. Hence, the electron level of the lowest intra-band-gap state leading to NIR emission would be situated about 1.98 eV above the valence band maximum (VBM) and about 0.40 eV below the conduction band minimum (CBM). It would be reasonable to ascribe this direct excitation to a transition from the VBM to intra-band-gap levels just below the CBM because based on DFT calculations of deep trap formation energies^39^ the presence of intra-band-gap electron trap levels below the CBM (at 1.98 eV) would be more likely than the existence of filled intra-band-gap levels just above the VBM (at 0.40 eV).

For CsPb(Br/Cl)_3_, the shift of the NIR excitation spectrum (Figure 1C) to higher energies relative to CsPbBr_3_ mimics that observed in the absorption spectra. In other words, excitation to intra-band-gap levels in both materials can lead to the population of NIR initial states. This blue shift of the NIR excitation spectrum is consistent with increasing Cl content (Figure S6, SI). Note, however, that the excitonic feature in the NIR excitation spectrum of CsPb(Br/Cl)_3_ is more prominent compared to its absorption spectrum and compared to the NIR excitation spectrum of CsPbBr_3_. It is known that the prominence of an excitonic peak at the absorption edge increases from iodide to bromide and chloride since materials with a wider band gap show a more pronounced excitonic peak, with exciton binding energies larger than the room-temperature thermal energy *k*_B_*T* = 0.026 eV.^40,41^ This means that for CsPbBr_3_ and CsPb(Br/Cl)_3_ the various excited-state species have a different relative importance in the pathway leading to NIR emission and that, although the NIR emission spectra of CsPbBr_3_ and CsPb(Br/Cl)_3_ are the same, the exciton and charge carrier dynamics associated with the NIR luminescence may be different. This can be expected based on the difference in exciton binding energy between the pure CsPbBr_3_ and mixed-halide CsPb(Br/Cl)_3_, with excitons being energetically more stable in the latter, leading to a larger separation in energy between the exciton and band gap absorption.^34,42,43^ The excitation spectrum of the NIR emission suggests that for CsPbBr_3_, free electrons and holes generated by band gap absorption would be the main species leading to NIR emission, with excitons playing a minor role. Conversely, for CsPb(Br/Cl)_3_, light absorption in the exciton band would be more efficient in generating NIR emission, whereas direct excitation of the free carrier would be a minor process.

Interestingly, analogous to that of CsPbBr_3_, the longest excitation wavelength that would still induce the NIR emission of CsPb(Br/Cl)_3_ is ca. 625 nm. This means that, although the band gap of CsPb(Br/Cl)_3_ is 0.07 eV higher than that of CsPbBr_3_, the NIR trap site energy seems to be at the same energy level as in CsPbBr_3_, namely, 1.98 eV above the VBM. Thus, the results from room-temperature steady-state absorption, NIR emission, and excitation spectroscopy allow us to hypothesize that the NIR emission of CsPbBr_3_ and CsPb(Br/Cl)_3_ originates from a radiative transition between an electron and a hole trapped by intra-band-gap levels. Such geminate electron and hole traps would be responsible for low-energy broad-band emission between donor and acceptor levels.^44^

To further develop the energetic picture described above, the visible and NIR emission spectra were acquired while varying the temperature between 294 and 361 K (Figure 2A and 2C and Figure S8, SI). In this temperature range, CsPbBr_3_ retains an orthorhombic crystal structure.^36,45,46^ The temperature-dependent emission spectra were then analyzed using eq 1, for which a mathematical deduction is provided in the Supporting Information (SI)
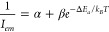
1in which *I*_*em*_ is the integrated PL intensity, α and β are parameters related to the rate constants for radiative and nonradiative relaxation (including both nonradiative recombination of the excitons and trapping into deep traps), Δ*E*_*a*_ is the activation energy for nonradiative relaxation, *k*_*B*_ is the Boltzmann constant, and *T* is absolute temperature. The temperature dependence of the visible emission yields activation energies of (0.05 ± 0.02) eV for CsPbBr_3_ and (0.06 ± 0.01) eV for CsPb(Br/Cl)_3_. Similar activation energies have been reported for the effect of temperature on the quenching of luminescence (0.06 eV)^47^ and on luminescence blinking (0.05–0.13 eV)^48^ in perovskite crystals. It has also been theoretically pointed out that low-energy phonons of around 0.02 eV play an important role in nonradiative relaxation through midgap traps.^49^ The observed activation energy can therefore be ascribed to the energy required to split emissive excitons into free carriers or to promote phonon modes to induce the nonradiative decay of excitons.

**Figure 2:**
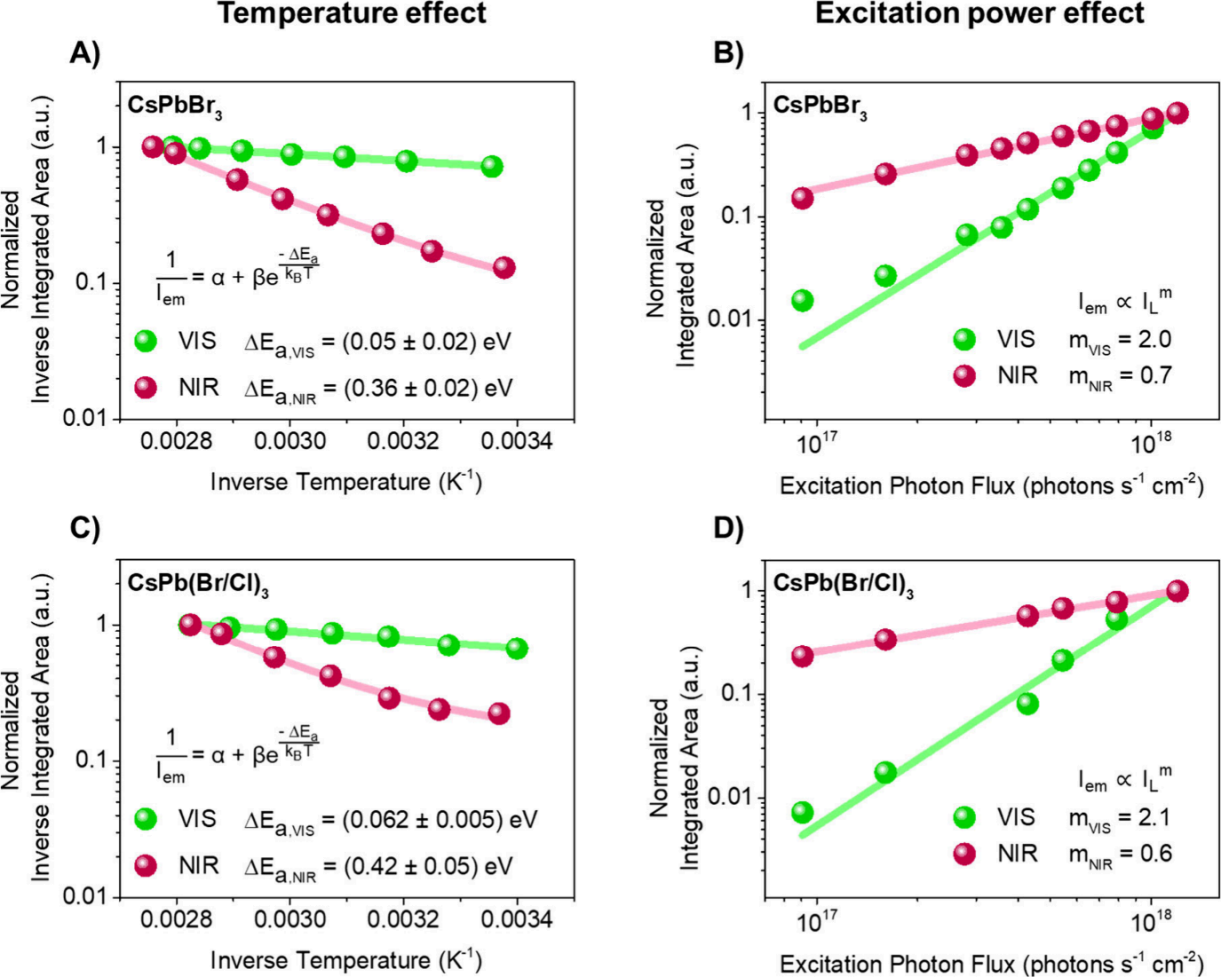
Temperature and excitation power dependence of the VIS and NIR emission of CsPbBr_3_ and CsPb(Br/Cl)_3_. A) Arrhenius plots of inverse integrated VIS and NIR emission intensity versus inverse temperature for CsPbBr_3_. B) Log–log plots of integrated VIS and NIR emission intensity versus excitation photon flux for CsPbBr_3_. C) Arrhenius plots of inverse integrated VIS and NIR emission intensity versus inverse temperature for CsPb(Br/Cl)_3_. D) Log–log plots of integrated VIS and NIR emission intensity versus excitation photon flux for CsPb(Br/Cl)_3_.

By contrast, increasing the temperature has a major impact on the NIR emission intensity, leading to a considerably higher activation energy of (0.36 ± 0.02) eV. The value of the latter activation energy can be physically interpreted in terms of the Schön-Klasens model.^50−52^ In this model, Δ*E*_*a*_ is considered to be an ionization energy or activation energy of detrapping for the species that contribute to the emission. For instance, for an electron bound at an electron trap, Δ*E*_*a*_ is the energy needed to thermally excite the electron to the conduction band, where it can diffuse to the traps associated with nonradiative decay of the VIS emission. The excitation spectra of the NIR emission suggest that the electron level of the lowest intra-band-gap state leading to NIR emission is found to be around 0.40 eV below the CBM. If the thermal deactivation of the NIR emission starts from this deep electron trap level, then the primary nonradiative relaxation channel could indeed involve the excitation of an electron from this trap level to the CBM. In this framework, the visible and NIR emission of CsPbBr_3_ microcrystals appear to have a common nonradiative relaxation channel. Although for CsPb(Br/Cl)_3_ the band gap is wider (2.45 eV), NIR excitation spectra demonstrate that the lowest initial excited state leading to NIR emission would have approximately the same energy as for CsPbBr_3_ (ca. 1.98 eV), which results in an energy level ca. 0.47 eV below the CBM of CsPb(Br/Cl)_3_. This energetic picture is corroborated by temperature-dependent NIR emission spectra of CsPb(Br/Cl)_3_ (Figure S9, SI), from which a higher activation energy for thermal quenching of (0.42 ± 0.05) eV was obtained compared to CsPbBr_3_. Hence, these findings on the activation energies are consistent with the idea that the lowest excited state leading to the NIR emission involves the excitation of electrons from the VBM to an intra-band-gap level about 1.98 eV above the VBM, pointing out that the same deep intra-band-gap levels are responsible for the NIR emission in CsPbBr_3_ and CsPb(Br/Cl)_3_. The nonradiative decay of the NIR emission then involves the excitation of an electron from this intra-band-gap level to the CB where it can diffuse to recombination centers, probably related to mid-band-gap states.^49^ Upon decreasing the temperature from 300 to 100 K, the NIR emission intensity increases substantially (by approximately a factor of 27) due to the suppression of nonradiative relaxation at low temperature (Figure S10, SI). Hence, the PLQY of the NIR emission is enhanced from 0.3% at room temperature to approximately 8% at 100 K. These results show that controlling the temperature allows tuning the efficiency of the NIR emission, which is of great significance for optimizing the performance of CsPbBr_3_ microcrystals as downconverting material in NIR emitting devices.

Besides the effect of temperature, the effect of excitation power on the VIS and NIR emission intensities (at room temperature) was investigated (Figure 2B and 2D and Figures S11 and S12, SI). The plots of the integrated area of the VIS and NIR emission spectra versus the excitation power could be fitted with a power law (eq 2)
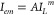
2in which *I*_*em*_ is the integrated PL intensity, *I*_*L*_ is the excitation photon flux (in photons s^–1^ cm^–2^), *m* is the power law exponent, and *A* is the amplitude. The power law exponent *m* for the VIS emission is found to be 2.0 for CsPbBr_3_ and 2.1 ± 0.1 for CsPb(Br/Cl)_3_, which is similar for both materials and in agreement with the value for direct band gap or exciton-mediated radiative recombination combined with efficient trapping of the electrons in deep traps acting as recombination centers (1 < *m* < 2).^53−55^ An exponent below 1 suggests a free-to-bound or radiative donor-to-acceptor transition which resembles radiative recombination^44^ between a trapped electron and a trapped hole in close proximity. The exponent for the NIR emission was found to be 0.7 for CsPbBr_3_ and 0.6 for CsPb(Br/Cl)_3_, hence the NIR power dependence is comparable for both materials and in agreement with the transition between an electron and a hole trapped in neighboring defect sites.^44,56,57^

Figure 3A presents the hypothetical energy diagram of CsPbBr_3_ and CsPb(Br/Cl)_3_ based on room-temperature absorption, emission, and excitation spectroscopy, combined with the results from temperature- and power-dependent luminescence experiments. Here, the NIR emission is represented as the result of the radiative donor–acceptor pair transition between an electron and a hole trapped at defect sites in close proximity. When cautiously comparing our observations with the defect charge transition levels calculated by Kang et al. using DFT^39^ (Figure 3B), it becomes clear that trap levels associated with Pb^2+^ interstitial and Pb^2+^/Br^–^ antisite defects, which can exist in CsPb(Br/Cl)_3_ as well as in CsPbBr_3_, are in an energy range compatible with our proposed electron and hole trap levels. Such deep-level traps have been observed even in melt-grown bulk CsPbBr_3_ crystals.^58^ Furthermore, it should be noted that no NIR emission was observed from CsPbBr_3_ nanocrystals synthesized in our laboratory.^59^ This suggests that the density of the defects leading to NIR emission is low. A detailed study into the effect of crystal size on the NIR emission intensity is being prepared.^60^

**Figure 3:**
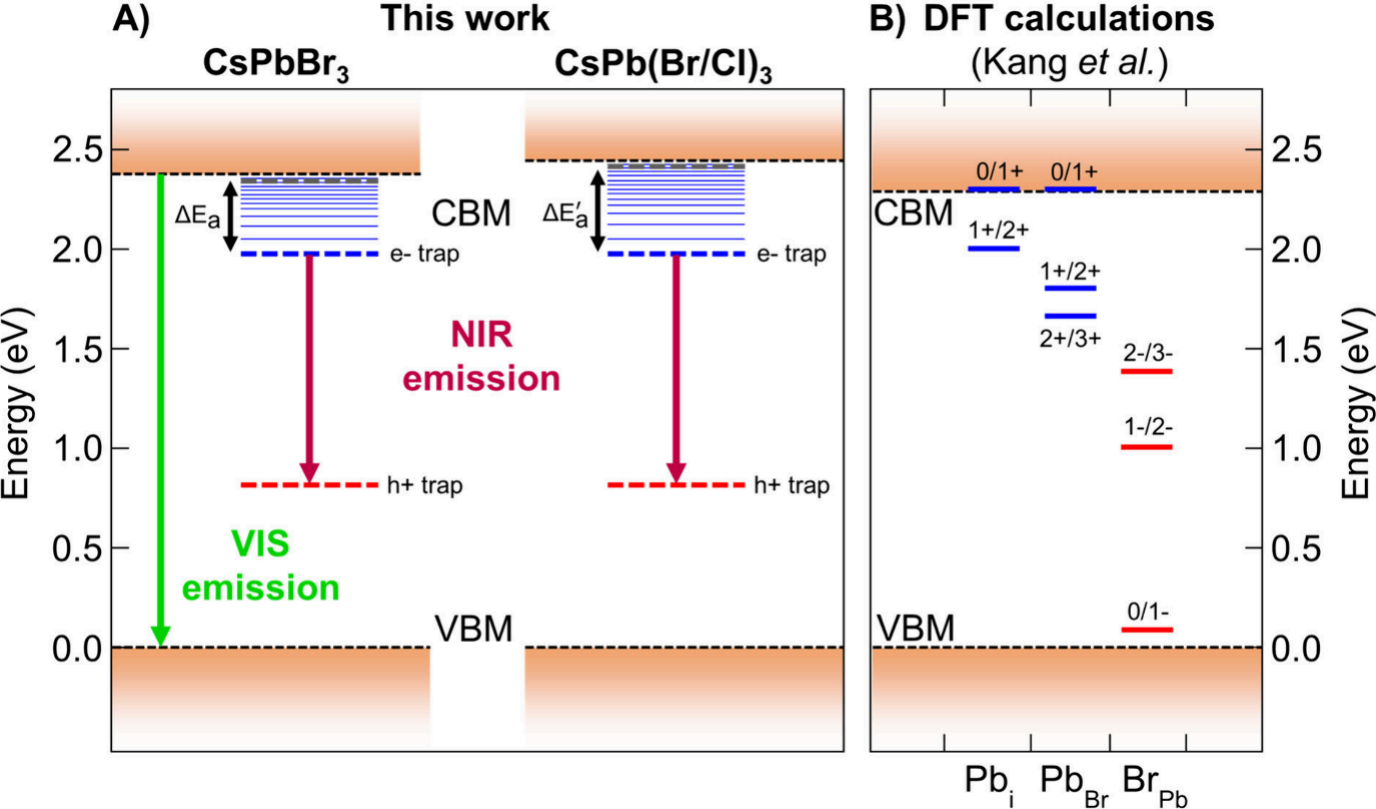
Energy diagram of CsPbBr_3_ and CsPb(Br/Cl)_3_ NIR emission. A) Experimentally deduced energy levels. B) Comparison with DFT calculations by Kang et al.^39^

To gain insight into the carrier dynamics associated with the VIS and NIR emission, time-resolved PL experiments were conducted in the picosecond to nanosecond time window, employing two different techniques.

First, PL decays of the visible (det. 600 nm) and NIR (det. 1150 nm) emission were obtained in the 200 ns time window using nanosecond time-resolved photoluminescence spectroscopy (nsPL) involving an InGaAs photodiode^61^ coupled to an oscilloscope (Figure 4A). The decays were analyzed by fitting to a multiexponential decay (eq 3),
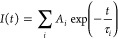
3in which *I*(*t*) is the PL intensity at time *t*, τ_*i*_ is the decay (or rise) time, and *A*_*i*_ is the corresponding amplitude. The resulting parameters are listed in Table S1 (SI). For both CsPbBr_3_ and CsPb(Br/Cl)_3_, the luminescence decays of the VIS and NIR emissions could be analyzed as a sum of two exponentials. Both decay times of the NIR emission (for CsPbBr_3_: 11 and 109 ns) are significantly longer than those obtained for the visible emission (for CsPbBr_3_: 2.7 and 29 ns). This difference in excited-state dynamics implies that the origin of the NIR emission of CsPbBr_3_ is not the same as that of the visible emission. The VIS emission is usually attributed to band-to-band radiative recombination of free carriers and free excitons^40,53−55^ or to trap-assisted radiative recombination of localized excitons in shallow defects situated close (within ±0.10 eV) to the CBM or VBM.^62−66^ Considering the interpretation of the luminescence decays on MHP nanocrystals,^67^ the short decay time could be a combination of the rate constants for carrier trapping and recombination both in shallow and deeper traps while the long decay time can be attributed to delayed fluorescence generated by detrapping of free carriers trapped in the shallow traps.^67^ Since the NIR excitation spectra indicated that excitation of electrons to deeper energy levels of up to 0.31 eV below the CBM could generate the NIR emission, it is plausible that upon band gap excitation the population of these trap levels also leads to the NIR emission. Hence, the time dependence of the NIR emission then rather reflects that of the population of these traps and the hole traps involved in the NIR emission (Figure 3). Besides, it should be noted that CsPbBr_3_ and CsPb(Br/Cl)_3_ have slightly different NIR decay times, although their steady-state NIR emission spectra are identical.

**Figure 4:**
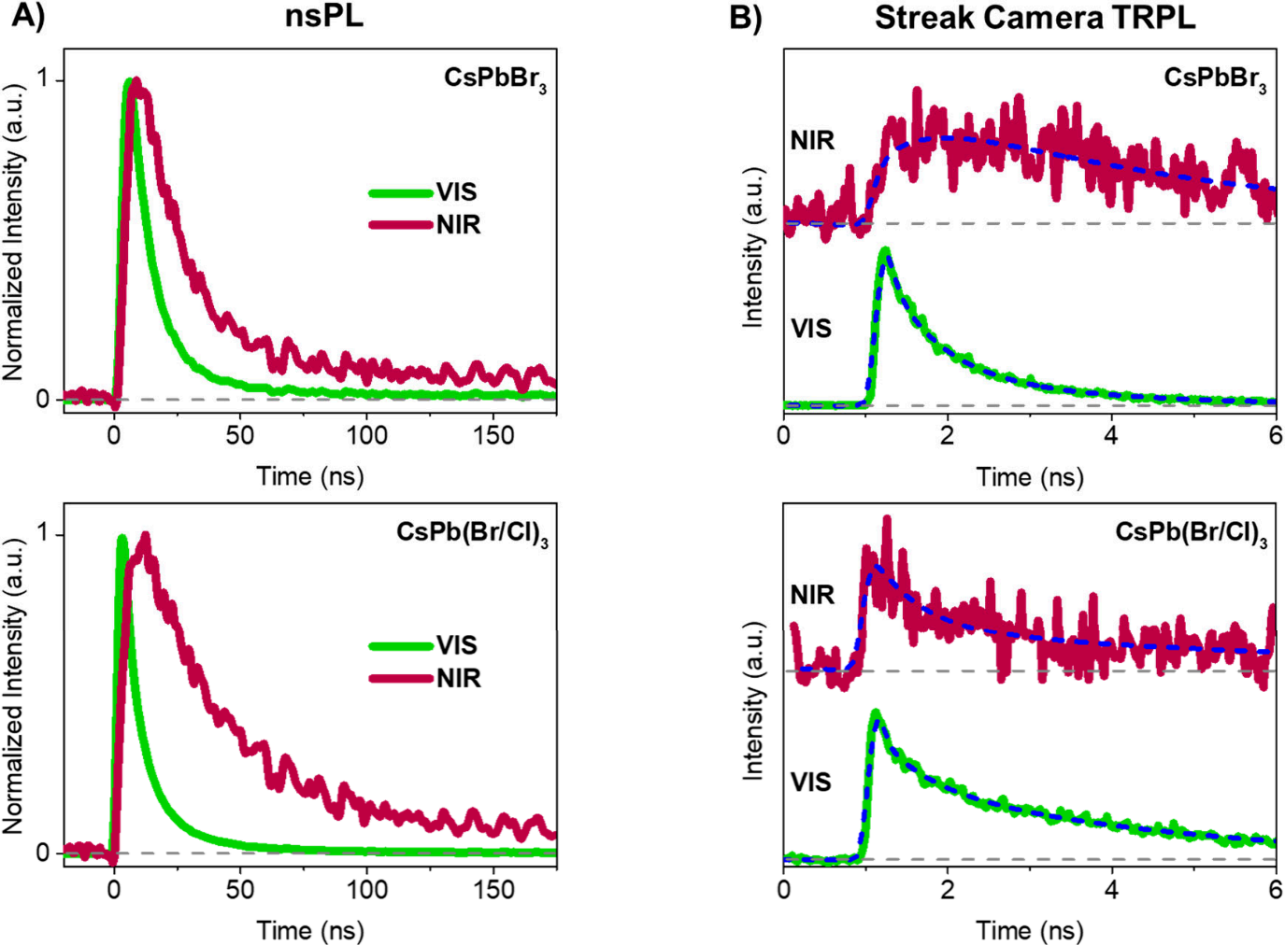
Time-resolved luminescence traces recorded at RT. A) VIS (det. 600 nm) and NIR (det. 1150 nm) PL decay traces of CsPbBr_3_ (top) and CsPb(Br/Cl)_3_ (bottom) measured with nsPL spectroscopy (exc. 532 nm). B) VIS (det. 490–530 nm) and NIR (det. 794–834 nm) PL decay traces of CsPbBr_3_ (top) and CsPb(Br/Cl)_3_ (bottom) measured with streak camera (exc. 445 nm) (the dashed gray lines indicate zero intensity).

To further investigate the dynamics related to the population of the free carriers and the deep trap levels involved in the NIR emission, time-resolved PL traces were obtained in a 10 ns time window using a picosecond streak camera system^68−70^ (Figure 4B). The data were analyzed by fitting the VIS and NIR signals globally to a multiexponential decay (eq 3), and the relevant fitting parameters are summarized in Table S2 (SI). In this case, the decays were analyzed with four exponential components. For CsPbBr_3_, the two longest decay times of the visible emission (2.2 and 18 ns) are in the same range as those obtained using nsPL (2.7 and 29 ns). Furthermore, the visible emission of CsPbBr_3_ shows two subnanosecond decay components that match the rise components of the NIR emission. The NIR emission decays on the nanosecond time scale, with the longest decay time (18 ns) which is of the same order of magnitude as the major component of the decay of the NIR emission obtained with nsPL (11 ns). The inability to detect the 109 ns component of the NIR emission obtained using nsPL is due to a combination of the short time window of the streak camera system used and the small amplitude of the latter component. These findings allow us to put forward the following excited-state relaxation mechanism from the states yielding the visible emission to the initial states leading to NIR emission. After band-to-band excitation (streak camera experiments) or the photogeneration of excitons (nsPL experiments), free electrons are generated in the conduction band (along with holes in the valence band). Subsequently, with time constants of around 86 and 660 ps, the conduction band is being depopulated (given by the decay of the visible emission), and at the same time, the NIR initial state is being populated (given by the rise in the NIR emission). The 660 ps component is in agreement with previous studies which show that a decay time of ca. 500 ps can be ascribed to the relaxation of free carriers to deep trap sites by means of a hopping process^71^ involving a series of spontaneous trapping–detrapping steps (processes 3 and 5 in Figure S13) in which the free carriers equilibrate among deep traps, shallow traps, and the conduction band.^71−76^ In this framework, the 86 ps component would correspond to either equilibration between free carriers and excitons or to the time constant of the first trapping step (process 3 in Figure S13).^54,77,78^ For a fraction of the carriers trapped at a deep level, radiative recombination from this level by emitting NIR light can occur if there is a neighboring hole trap. In this picture, the 2.7 ns component obtained using nsPL would then correspond either to the detrapping of weakly trapped carriers (process 5) or the decay of the emission from shallow traps rather than to a combination of deep trapping and recombination (processes 6 and 8 in Figure S13) of free carriers or free excitons as suggested by the nsPL experiments above. Ascribing the 2.7 ns component to the detrapping of weakly trapped carriers (process 5) is also more compatible with the extremely low PLQY of the visible emission (5 × 10^–5^). This would mean that the detrapping of weakly trapped carriers (process 5) contributes to both the 660 and 86 ps components due to the fact that there is a distribution of the energy levels of the shallow traps.^67^

In conclusion, we thoroughly examined the NIR luminescence generated by undoped, all-inorganic CsPbBr_3_ and CsPbBr_2.6_Cl_0.4_ microcrystals. We have pointed out that that NIR emission is a relatively important relaxation process with an NIR PLQY in the range of 0.3% and infer that this radiative transition originates from intrinsic deep electron and deep hole trap sites. The excited-state dynamics of the NIR emission of CsPbBr_3_ is governed by a 660 ps rise component ascribed to the population of deep NIR initial states through a trapping–detrapping mechanism. Our work opens the possibility for the development of NIR-emitting light sources based on CsPbBr_3_ microcrystals as stable, low-cost downconverting phosphors when excited by visible light, with possible applications in biological imaging as the emission overlaps with the second biological NIR window (NIR-II) (1000–1400 nm).^25,26^ Our results show that cooling highly improves the PLQY of the NIR emission, which is of major importance to the optimalization of potential NIR emitting devices. Moreover, since the NIR emissive channel involving deep-level traps is not negligible, the fundamental insights regarding this additional pathway, which in the case of solar panels and visible-range LEDs would lead to undesirable carrier recombination, can contribute to improving the efficiency of these technologies.

## Experimental Methods

Experimental details about the synthesis and characterization techniques are provided in the Supporting Information (SI).

## Supporting Information Available

Synthesis methods; detailed measurement procedures; additional experimental results for CsPbBr_3_ and CsPb(Br/Cl)_3_ including SEM images; XRD patterns, VIS and NIR microscopy images; electronic band gap estimation; VIS and NIR photoluminescence spectra at different excitation wavelengths, temperatures, and excitation intensities; NIR excitation spectra for different Cl contents; fitting parameters for the time-resolved PL data; and a schematic energy diagram depicting the photophysical processes after excitation in CsPbBr_3_ microcrystals (PDF)
