# Supplementary material for: NIR Luminescence from Deep-Level Traps in CsPbBr3 Microcrystals
Source: J Phys Chem Lett. 2025 Mar 31;16(14):3491–500. doi: 10.1021/acs.jpclett.5c00545 (PMC12053942; doi:10.1021/acs.jpclett.5c00545)
Supplement: Supplementary file 1 — jz5c00545_si_001.pdf [file jz5c00545_si_001.pdf]

Supporting Information for

NIR Luminescence from Deep-Level Traps in $CsPbBr_3$ Microcrystals

Jonathan Vandenwijngaerden [1], Bapi Pradhan [1], Bob Van Hout [1], Eduard Fron [1], Yasuyuki Araki [2], Xianjun Zhang [3,4], Yutaka Shibata [3], Dario Santantonio [5], Roger Bresoli-Obach [1,5], Santi Nonell [5], Haifeng Yuan [1,6], Jialiang Xu [7], Mark Van der Auweraer [1], Maarten Roeffaers [8], Johan Hofkens [1,9], Hiroshi Fukumura [1,3] and Elke Debroye* [1]*

[1] Molecular Imaging and Photonics, Department of Chemistry, KU Leuven, Celestijnenlaan 200F, 3001 Leuven, Belgium

[2] Institute of Multidisciplinary Research for Advanced Materials, Tohoku University, Katahira 2-1-1, Aoba-ku, Sendai, Japan

[3] Department of Chemistry, Graduate School of Science, Tohoku University, 6-3 Aramaki Aza-Aoba, Aoba-ku, Sendai, Japan

[4] Department of Chemistry, Massachusetts Institute of Technology, Cambridge, Massachusetts 02139, United States

[5] AppLightChem, Institut Químic de Sarrià, Universitat Ramon Llull, Via Augusta 390, Barcelona, Catalunya 08017, Spain

[6] Yongjiang Laboratory, 1792 Cihai South Road, Ningbo 315202, China

[7] School of Materials Science and Engineering, Tianjin Key Laboratory of Metal and Molecular Materials Chemistry, Frontiers Science Center for New Organic Matter, Nankai University, Tongyan Road 38, Tianjin 300350, China

[8] cMACS, Department of Microbial and Molecular Systems, KU Leuven, Celestijnenlaan 200F, 3001 Leuven, Belgium

[9] Max Planck Institute for Polymer Research, Ackermannweg 10, 55128 Mainz, Germany

Corresponding Author

* To whom all correspondence should be addressed: Elke Debroye (email: elke.debroye@kuleuven.be), Johan Hofkens (email: johan.hofkens@kuleuven.be)

Contents

Synthesis Procedures....4
Characterization Procedures....6
Results and Discussion....13
References....30

Synthesis Procedures

Reagents

The precursor chemicals were purchased commercially as follows: CsBr (99%, Alfa Aesar), $PbBr_2$ (98%, Sigma Aldrich), $PbCl_2$ (99%, Thermo Scientific), HBr (48 wt%, Thermo Scientific), polystyrene (average molar weight 260'000, Acros Organics). All reagents and solvents were used without further purification.

Synthesis of $CsPbBr_3$ Microcrystals

$CsPbBr_3$ microcrystals are synthesized at room temperature under ambient conditions, according to the protocol of Huang *et al.*[1] First, stoichiometric amounts of CsBr (0.633 mmol) and $PbBr_2$ (0.633 mmol) are dissolved in 1 mL dimethyl sulfoxide (DMSO) by vigorous stirring for 30 minutes until a semitransparent solution is obtained. Subsequently, 1 mL pure HBr is added dropwise to the precursor solution under constant stirring. An orange precipitate appears instantly, marking the formation of CsPbBr3 microcrystals. The product is then centrifuged (8500 rpm, 2 min) and washed twice with 1 mL ethanol. Finally, the powder is dried in vacuum at room temperature. The powder samples are kept in close vials and can be employed for spectroscopic experiments in ambient air for months without showing signs of degradation.

Synthesis of $CsPb(Br/Cl)_3$ Microcrystals

$CsPbBr_{2.6}Cl_{0.4}$ microcrystals with mixed halide content ($CsPb(Br/Cl)_3$) are synthesized starting from CsBr (0.633 mmol), $PbBr_2$ (0.158 mmol) and $PbCl_2$ (0.475 mmol) and following the same protocol as described for the pure $CsPbBr_3$ microcrystals. Although the Br:Cl ratio in the precursors is 1:1, the mixed halide perovskite microcrystals are expected to be more rich in Br due to the likely formation of CsCl which has a poor solubility in DMSO[2], and because HBr is used to precipitate the crystals. The powder samples are kept in close vials and can be employed for spectroscopic experiments in ambient air for months without showing signs of degradation.

Preparation of $CsPbBr_3$ Microcrystals Film for NIR Microscopy

First, a solution of 100 mg polystyrene in 1 mL toluene is prepared. Then, 5 mg of $CsPbBr_3$ MCs powder is added. The mixture is stirred until a homogeneous suspension is obtained. Finally, a film is prepare by spin coating the suspension on a glass slide.

Characterization Procedures

X-ray Diffraction

X-ray diffraction (XRD) patterns were measured on a Malvern PANalytical Empyrean diffractometer with a PIXcel3D solid-state detector using a Cu anode (Cu $K_{\alpha 1}$: 1.5406 Å; Cu $K_{\alpha 2}$: 1.5444 Å). The powder samples were loaded onto a 96-well sample holder and X-ray diffractograms were recorded at room temperature in a transmission geometry within a 1.3-45° 2θ range using a step size of 0.013°.

Scanning Electron Microscopy and Energy-Dispersive X-Ray Spectroscopy

Scanning electron microscopy (SEM) and energy-dispersive X-ray spectroscopy (EDX) measurements were recorded on a FEI-Q FEG250 instrument equipped with an EDAX detector.

Diffuse Reflectance Spectroscopy

Diffuse reflectance spectra of the powders in quartz cuvettes (1 mm path length) were recorded on a PerkinElmer Lambda 950 UV-visible spectrophotometer equipped with an integrating sphere. $BaSO_4$ powder was used as white reference. The diffuse reflectance spectra were converted to absorption equivalent spectra $\alpha(\lambda)$ using the Kubelka−Munk function $F(R_\infty)$:[3]

$$\alpha(\lambda) = F(R_\infty) = \frac{K}{S} = \frac{(1\; -\; R_\infty)^2}{2R_\infty} \qquad \text{(S1.1)}$$

With R_∞ absolute reflectance at wavelength λ, K absorption coefficient, S scattering coefficient.

Estimation of Electronic Bandgap

As there are excitonic bands in the Kubelka-Munk equivalent absorption spectrum, a Tauc plot cannot be used to calculate the electronic bandgap E_g, as this would lead to systematic underestimation of E_g. Instead, the bandgap can be estimated according to the following equation:[4]

$$E_g = E_x + E_{b,x} \tag{S2.1}$$

With E_g the electronic bandgap energy, E_x the energy of the excitonic absorption peak and $E_{b,x}$ the exciton binding energy, which amounts to 33 meV for bulk $CsPbBr_3$[5,6].

Steady-State Photoluminescence Spectroscopy

Emission and excitation spectra of the powders in the visible (VIS) detection region (400-800 nm) were measured on an Edinburgh Instruments FLS980 spectrofluorometer with PMT detector (Hamamatsu R928P multialkali photocathode). The emission spectra were corrected for the wavelength dependence of the detection channel throughput. Using a quantum counter, the excitation spectra were corrected for the wavelength dependence of the intensity of the excitation light as well as for temporal fluctuations of this intensity.

Emission and excitation spectra of the powders in the near-infrared (NIR) detection region (680-1660 nm) were measured on a PTI QuantaMaster 50 spectrofluorometer with near-infrared detector (Judson J22 InGaAs photodiode). The emission spectra were corrected for the wavelength dependence of the detection channel throughput. Using a quantum counter, the excitation spectra were corrected for the wavelength dependence of the intensity of the excitation light.

Temperature-dependent emission spectra in the temperature range 286-370K were measured on the aforementioned Edinburgh (VIS emission) and PTI (NIR emission) spectrofluorometers by means of a cuvette holder connected to a temperature control unit. The effective temperature of the

sample is gauged by placing a temperature sensor directly at the surface of the cuvette containing the sample.

Estimation of Near-Infrared Quantum Yield

The photoluminescence quantum yield of the NIR emission was estimated using the following equation:[7]

$$\phi_{em,680-1600nm} = \phi_{em,680-800\,nm} * \frac{I_{680-1600\,nm}}{I_{680-800\,nm}} \tag{S3.1}$$

With $\phi_{em,680-1600nm}$ the total PLQY of the NIR emission, $\phi_{em,680-800\,nm}$ the absolute partial PLQY measured with the integrating sphere technique on the abovementioned Edinburgh FLS980 spectrofluorometer using $BaSO_4$ as a reference, $I_{680-1600\,nm}$ the total integrated area of the NIR emission band, $I_{680-800\,nm}$ the integrated area of the NIR emission in the 680-800 nm wavelength range. Here, the integrated intensities *I* are expressed in terms of photonic quantities (photons per second) to take into account the different energies of photons with different wavelengths.[8] The partial PLQY $\phi_{em,680-800\,nm}$, as well as the PLQY of the visible emission, were measured at an excitation flux of ca. 10^{15} photons s^{-1} cm^{-2}.

Nanosecond Time-Resolved Photoluminescence Spectroscopy

Photoluminescence decays in the 200 ns time window were recorded using InGaAs photodiodes (PDs) coupled to an oscilloscope.[9] The laser beam from PL2210A (EKSPLA, 1 kHz, 25 ps, 532 nm) was split into two beams by a beam splitter, and one beam was directed to an InGaAs PD (G10899-01K, 400-1600 nm, Hamamatsu Photonics) for IRF data.[10] The other beam was directed to the sample and the emission scattered from the surface of the sample was detected by another InGaAs PD (G10899-01K, 400-1600 nm, Hamamatsu Photonics). The NIR component was then passed through two filters (O58 and RM100, Edmund Optics). The signals from both InGaAs PDs

were acquired as digital data after averaging 1000 events on a digital oscilloscope (LeCroy WaveSurfer 4024 HD). The time resolution, based on the FWHM of the instrumental response function (IRF), was around 6 ns. The results of the measurements were analyzed by the software (DecayFit version 1.4). The quality of the fit was monitored by the value of χ^2.

Streak Camera Time-Resolved Spectroscopy

Photoluminescence decays in the 10 ns time window were measured using a picosecond streak camera system. The excitation occurred at 445 nm by pulses of a duration of ca. 150 fs and a flux of 1.8 kW/cm². The repetition rate amounted to 80 MHz. The time resolution, based on the FWHM of the IRF, was around 80 ps. The technical details of the setup are described in previous reports.[11–13] The data was analyzed by fitting to a multiexponential decay and the quality of the fit was monitored by the value of χ^2.

Estimation of Activation Energy for Nonradiative Relaxation

The photoluminescence quantum yield (PLQY, ϕ_{em}) is the ratio of the number of emitted photons to the number of absorbed photons.[14] An excited state is depopulated through radiative relaxation, with rate constant k_R, and nonradiative relaxation, with rate constant k_{NR}^{tot}. Since the quantum yield is equal to the fraction of excited states that decay via emission, it is given by:[15]

$$\phi_{em} = \frac{k_R}{k_R + k_{NR}^{tot}} \tag{S4.1}$$

The rate constant k_{NR}^{tot} represents all possible nonradiative decay processes. Since these processes can be either temperature-dependent or temperature-independent, it can be instructive to treat these contributions separately:

$$k_{NR}^{tot} = k_{NR}^{0} + k_{NR}(T) \tag{S4.2}$$

With k_{NR}^{0} rate constant for temperature-independent nonradiative relaxation and $k_{NR}(T)$ rate constant for temperature-dependent nonradiative relaxation. $k_{NR}(T)$ is dependent on the temperature according to the Arrhenius equation:

$$k_{NR}(T) = k'_{NR} e^{\frac{-\Delta E_a}{k_B T}} \tag{S4.3}$$

With k'_{NR} Arrhenius factor for the temperature-dependent nonradiative relaxation rate constant, ΔE_a activation energy for temperature-dependent nonradiative relaxation, k_B Boltzmann constant and T absolute temperature. Hence, equation (S1) becomes:

$$\phi_{em} = \frac{k_R}{k_R + k_{NR}^{0} + k'_{NR} e^{\frac{-\Delta E_a}{k_B T}}} \tag{S4.4}$$

This can be re-written as:

$$\frac{1}{\phi_{em}} = \left(1 + \frac{k_{NR}^{0}}{k_R}\right) + \left(\frac{k'_{NR}}{k_R}\right) e^{\frac{-\Delta E_a}{k_B T}} \tag{S4.5}$$

$$\frac{1}{\phi_{em}} = \alpha' + \beta' e^{\frac{-\Delta E_a}{k_B T}} \tag{S4.6}$$

The integrated intensity under the emission spectrum I_{em} is directly proportional to the quantum yield, so the equation finally becomes:

$$\frac{1}{I_{em}} = \alpha + \beta e^{\frac{-\Delta E_a}{k_B T}} \tag{S4.7}$$

If the inverse integrated area under the emission spectrum is plotted versus the inverse temperature, fitting the data to equation (S4.7) (equation (2) in the main text) allows to estimate the activation energy ΔE_a.

Furthermore, by dividing both sides in (S4.2) with k_R, an equation can be derived that relates the ratio of the total nonradiative and radiative rate constants to the experimental fitting parameters α' and β' of equation (S4.6):

$$\frac{k_{NR}^{tot}}{k_R} = \frac{k_{NR}^{0}}{k_R} + \frac{k_{NR}(T)}{k_R} \tag{S4.8}$$

$$\frac{k_{NR}^{tot}}{k_R} = \frac{k_{NR}^{0}}{k_R} + \frac{k'_{NR}}{k_R} e^{\frac{-\Delta E_a}{k_B T}} \tag{S4.9}$$

$$\frac{k_{NR}^{tot}}{k_R} = (\alpha' - 1) + \beta' e^{\frac{-\Delta E_a}{k_B T}} \tag{S4.10}$$

Microscopy

To measure NIR microscopy images, a 532 nm laser line (CPS532, Thorlabs) was used for widefield illumination by focusing the laser beam at the back focal plane of the objective lens. The excitation laser was filtered with a KG-5 filter (Schott) to remove any residual NIR component. The excitation irradiance was 7500 W/cm^2. The NIR emission was collected by an air-immersion NIR objective lens (NA 0.40, 20x, PAL-20-NIR-LC00, OptoSigma) and filtered with two long pass filters (900 and 950 nm; Thorlabs) to remove the green emission of the perovskite sample and the residual component of the excitation laser. The image was projected onto an InGaAs NIR sensitive camera (C14041-10U, Hamamatsu) using a tube lens (f = 200 mm Thorlabs).

VIS microscopy images under 532 nm excitation were recorded on a confocal laser scanning microscope (Olympus Fluoview FV1000). The data was processed using ImageJ software.

Results and Discussion

Structural and Morphological Characterization

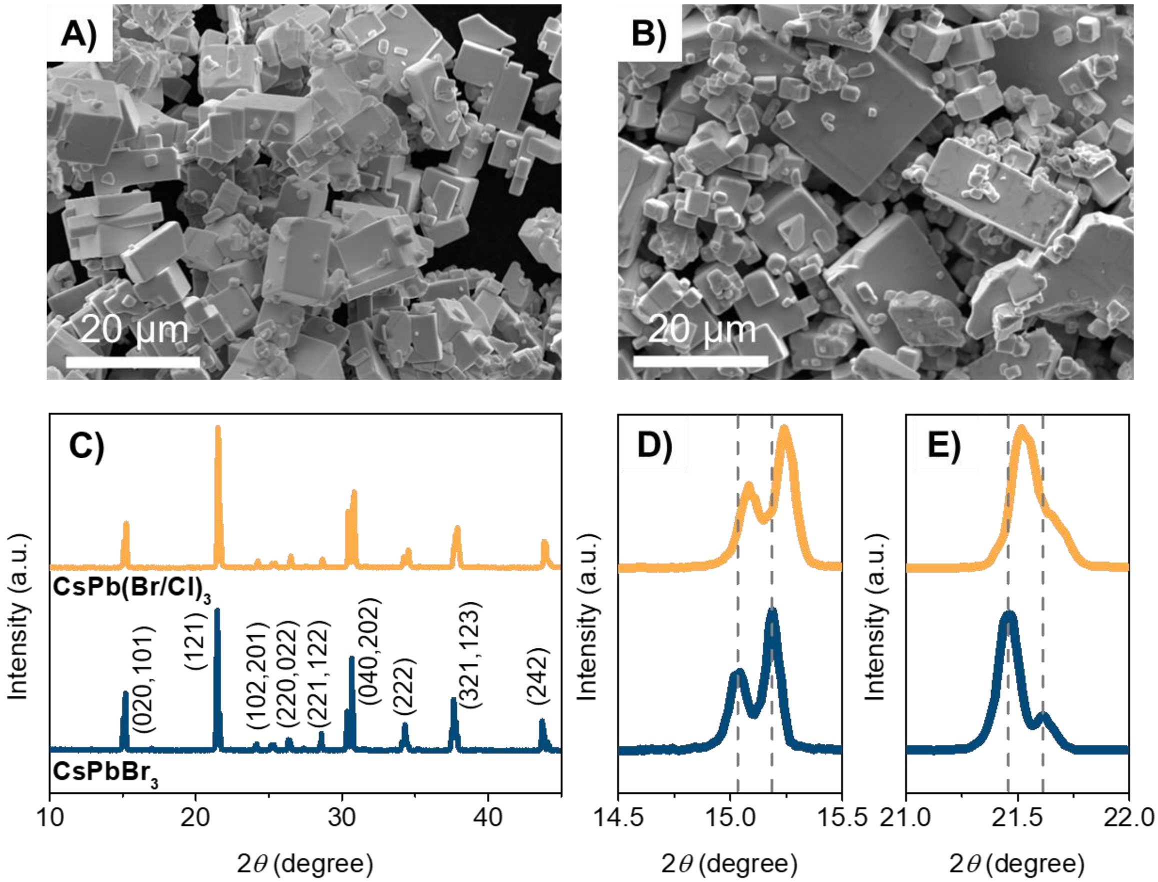

Figure S1. A) SEM image of $CsPbBr_3$ microcrystals; B) SEM image of $CsPb(Br/Cl)_3$ microcrystals; C) Powder XRD patterns of $CsPbBr_3$ and $CsPb(Br/Cl)_3$ microcrystals; D) Selected XRD peaks near $2\theta = 15°$; E) Selected XRD peaks near $2\theta = 21.5°$

Scanning electron microscopy (SEM) images (Figure S1A-B) indicate that the microcrystals have a cuboid shape with dimensions in the order of a few to tens of micrometers. The Br:Cl ratio of $CsPbBr_{2.6}Cl_{0.4}$ was quantified using energy-dispersive X-ray spectroscopy (EDX). This Cl content (~14%) is lower than what would be expected from equimolar amounts of Br and Cl in the precursors. Probably, this is due to the formation of CsCl which has a poor solubility in DMSO[2],

and due to the use of HBr to precipitate the microcrystals. By powder X-ray diffraction (XRD), the crystal structure of $CsPbBr_3$ was confirmed to be that of crystalline CsPbBr3 perovskite in the orthorhombic phase (Figure S1C). $CsPb(Br/Cl)_3$ exhibits the same dominant peaks as $CsPbBr_3$, although they are all shifted to higher 2θ values. This is consistent with the replacement of some bromine atoms by smaller chlorine atoms, causing a contraction of the crystal lattice and a shift of the XRD peaks to higher angles. Using Bragg's law, the approximate lattice parameter of $CsPb(Br/Cl)_3$ was found to be 0.3% smaller than that of $CsPbBr_3$, which is in accord with values reported for mixed-halide perovskites with similar Cl content.[16]

Microscopy

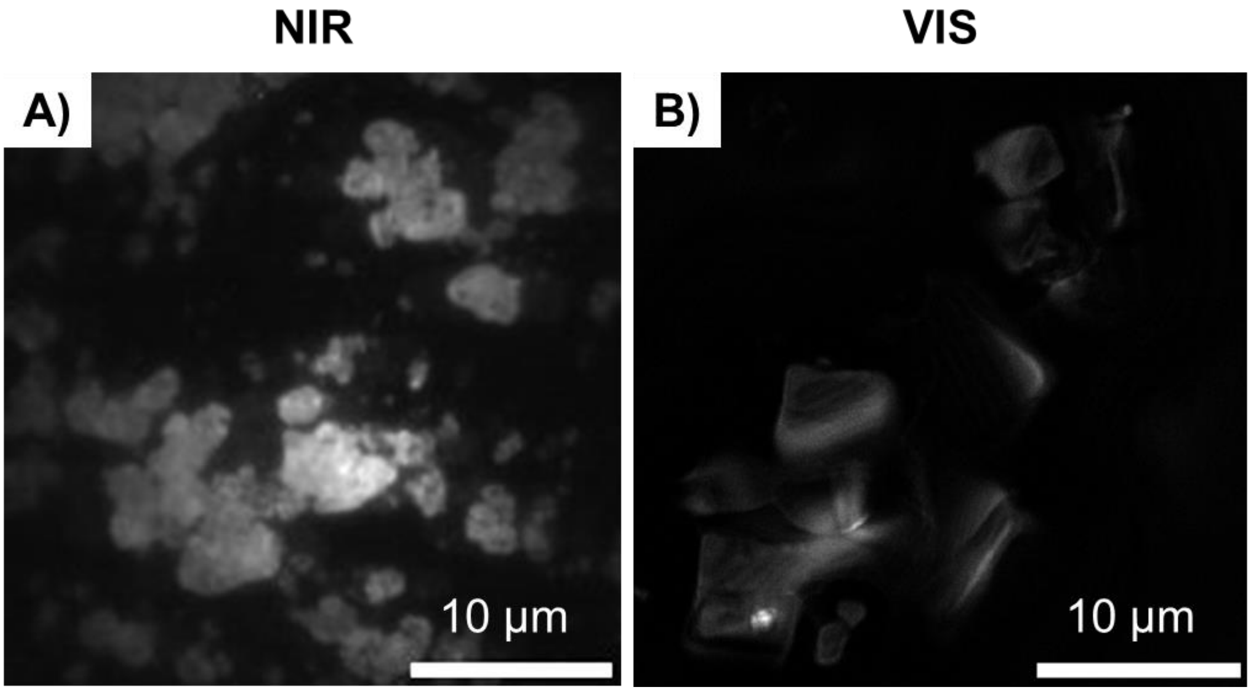

Figure S2. Emission images of $CsPbBr_3$ microcrystals dispersed in a polystyrene matrix (exc. 532 nm, I_{532} = 7500 W/cm^2). A) NIR image (det. 950-1700 nm); B) VIS image (det. 540-600 nm). The VIS and NIR emission images were measured from different locations using different setups.

Estimation of Electronic Bandgap

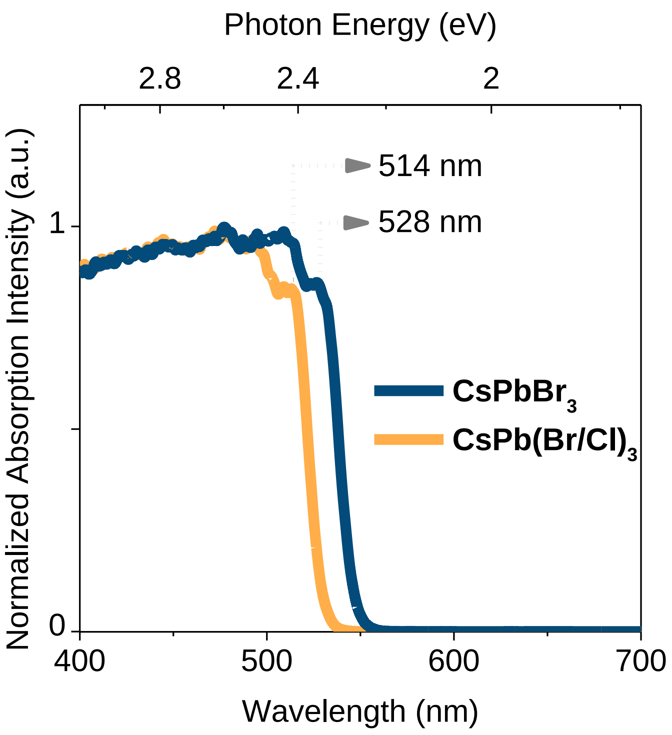

Figure S3. Kubelka-Munk equivalent absorption spectrum of $CsPbBr_3$ and $CsPb(Br/Cl)_3$ indicating the position of the excitonic peaks. The spectra are normalized to one at the maximum.

Using equation (S2.1), the maxima of the excitonic band which are situated at 2.348 eV (528 nm) and 2.412 eV (514 nm) for $CsPbBr_3$ and CsPb(Br/Cl) yield a bandgap at respectively 2.38 eV for $CsPbBr_3$ and 2.45 eV for $CsPb(Br/Cl)_3$. The bandgap found for $CsPbBr_3$ is close to the value of 2.40 eV calculated from the literature value of the exciton maximum (2.37 eV) at room temperature for vapor deposited layers of $CsPbBr_3$.[5]

Photoluminescence Spectra

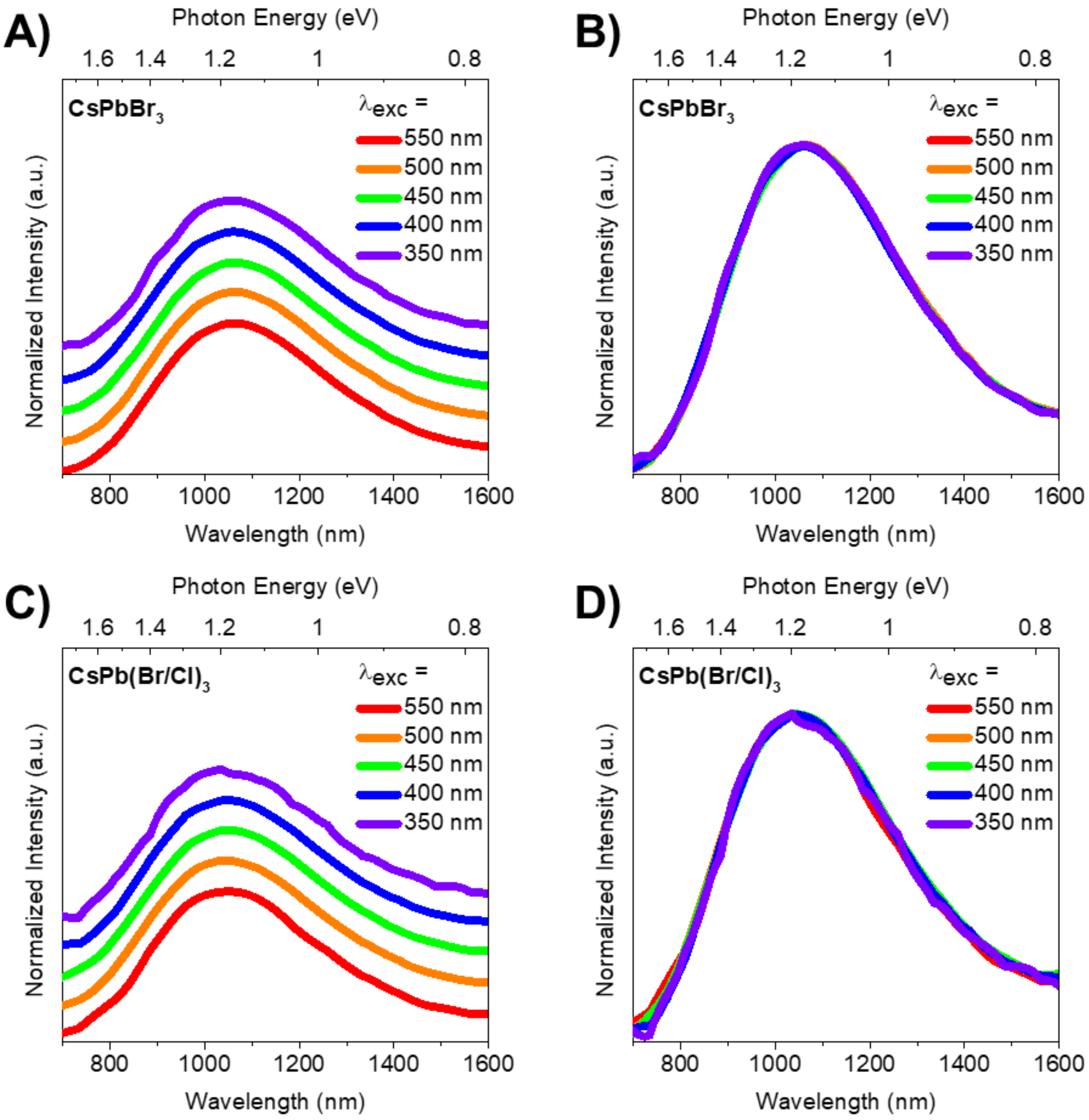

Figure S4. NIR emission spectra, normalized at the maximum, obtained for different excitation wavelengths. A) $CsPbBr_3$ stacked plot; B) $CsPbBr_3$ overlay plot; C) $CsPb(Br/Cl)_3$ stacked plot; D) $CsPb(Br/Cl)_3$ overlay plot

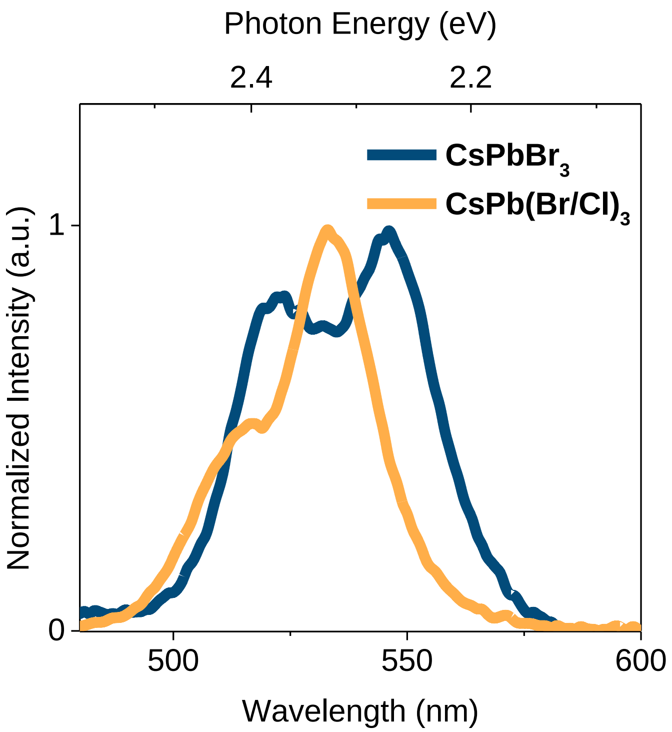

Figure S5. Normalized VIS emission spectra of $CsPbBr_3$ and $CsPb(Br/Cl)_3$ (exc. 400 nm)

The shift of the two peaks to higher energies, from 522 nm (2.38 eV) and 546 nm (2.27 eV) for $CsPbBr_3$ to 515 nm (2.41 eV) and 533 nm (2.33 eV) for $CsPb(Br/Cl)_3$, is due to the higher bandgap energy of $CsPb(Br/Cl)_3$ (2.45 eV) compared to $CsPbBr_3$ (2.38 eV), as was calculated from their absorption spectra (Figure S2, ESI). The blueshift in bandgap from $CsPbBr_3$ to $CsPb(Br/Cl)_3$ observed in our work is in agreement with literature data which shows that $CsPb(Br/Cl)_3$ has a larger bandgap than $CsPbBr_3$.[17] Moreover, the exciton binding energy in the Cl-containing material is higher (exciton binding energy in pure $CsPbCl_3$ amounts to 64 meV)[18] compared to that of pure $CsPbBr_3$ (33 meV)[5,6] Since the high energy PL peak corresponds to band-to-band and free exciton recombination, while the lower energy peak is usually ascribed to recombination of excitons bound to (shallow) trap sites,[19–21] the relative difference in peak shape between $CsPbBr_3$ and $CsPb(Br/Cl)_3$ can hence in part be attributed to the different contribution of bound excitons.

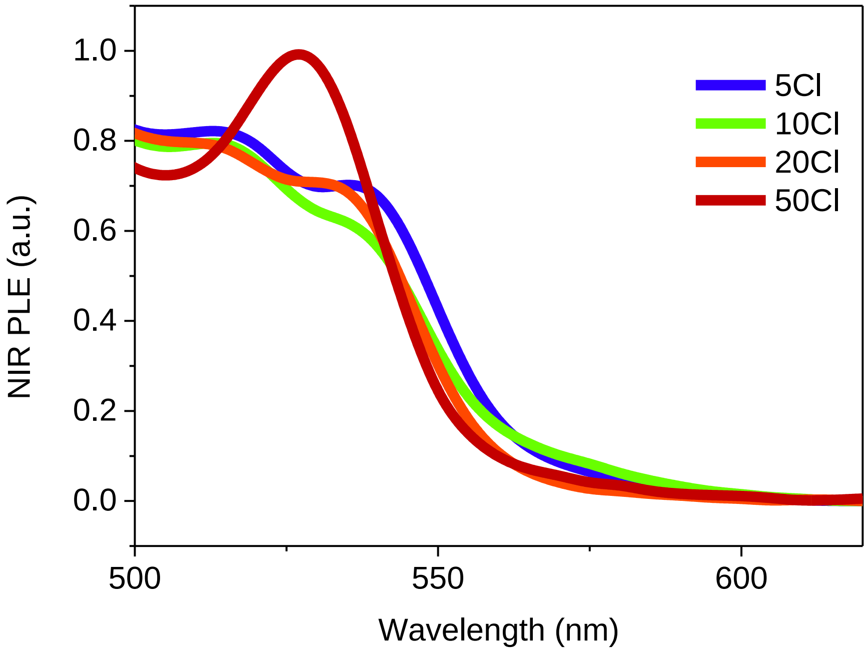

Figure S6. NIR excitation spectra (det. 1150 nm) for different Cl contents in the precursor mixture (5%, 10%, 20%, 50%). The $CsPbBr_{2.6}Cl_{0.4}$ in the manuscript corresponds to the 50% sample.

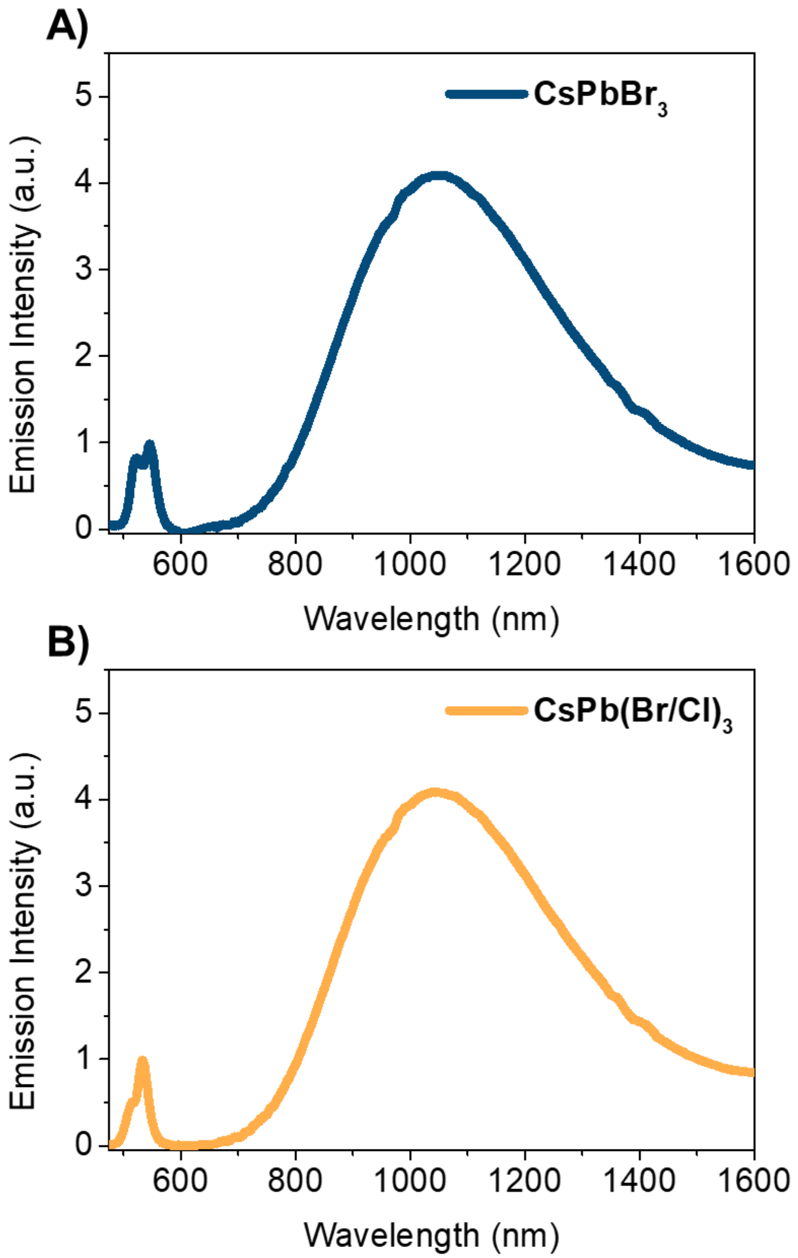

Figure S7. Combined and rescaled VIS (measured with PMT) and NIR (measured with InGaAs) emission spectra of A) $CsPbBr_3$; B) $CsPb(Br/Cl)_3$ (exc. 400 nm). The spectra were rescaled so that the area under the NIR emission spectrum is approximately 60 times that of the VIS emission spectrum (in wavelength scale), in correspondence with the ratio of the experimental VIS and NIR QY.

Time-Resolved Photoluminescence

Table S1. Analysis of the luminescence decays obtained via nanosecond photoluminescence spectroscopy (exc. 532 nm). The luminescence decays were fitted to a bi-exponential decay.

Sample	Detection wavelength (nm)	A_1	τ_1 (ns)	A_2	τ_2 (ns)	A_3	τ_3 (ns)	$\langle\tau\rangle$ (ns)
$CsPbBr_3$	600 (VIS)	0.94	2.7	0.06	29	/	/	4.3
	1150 (NIR)	/	/	0.89	11	0.11	109	22
$CsPb(Br/Cl)_3$	600 (VIS)	0.98	0.4	0.02	17	/	/	0.7
	1150 (NIR)	/	/	0.73	18	0.27	81	35

Table S2. Analysis of the luminescence decays obtained by a picosecond streak camera (exc. 445 nm). The luminescence decays were fitted to a quadruple exponential decay.

<table>
<tr><th>Sample</th><th>Detection wavelength (nm)</th><th>A_1</th><th>τ_1 (ns)</th><th>A_2</th><th>τ_2 (ns)</th><th>A_3</th><th>τ_3 (ns)</th><th>A_4</th><th>τ_4 (ns)</th><th>$\langle\tau\rangle$ (ns)</th></tr>
<tr><td rowspan="2">$CsPbBr_3$</td><td>490-530 (VIS)</td><td>0.50</td><td rowspan="2">0.086</td><td>0.43</td><td rowspan="2">0.66</td><td>0.07</td><td rowspan="2">2.2</td><td>/</td><td rowspan="2">18</td><td>0.5</td></tr>
<tr><td>794-834 (NIR)</td><td>-0.27 (rise)</td><td>-0.73 (rise)</td><td>0.64</td><td>0.36</td><td>7.9</td></tr>
<tr><td rowspan="2">$CsPb(Br/Cl)_3$</td><td>490-530 (VIS)</td><td>0.50</td><td rowspan="2">0.053</td><td>0.25</td><td rowspan="2">0.59</td><td>0.25</td><td rowspan="2">3.7</td><td>0.002</td><td rowspan="2">41</td><td>1.2</td></tr>
<tr><td>794-834 (NIR)</td><td>-1 (rise)</td><td>0.68</td><td>0.13</td><td>0.18</td><td>8.3</td></tr>
</table>

Temperature Dependent Photoluminescence Spectra

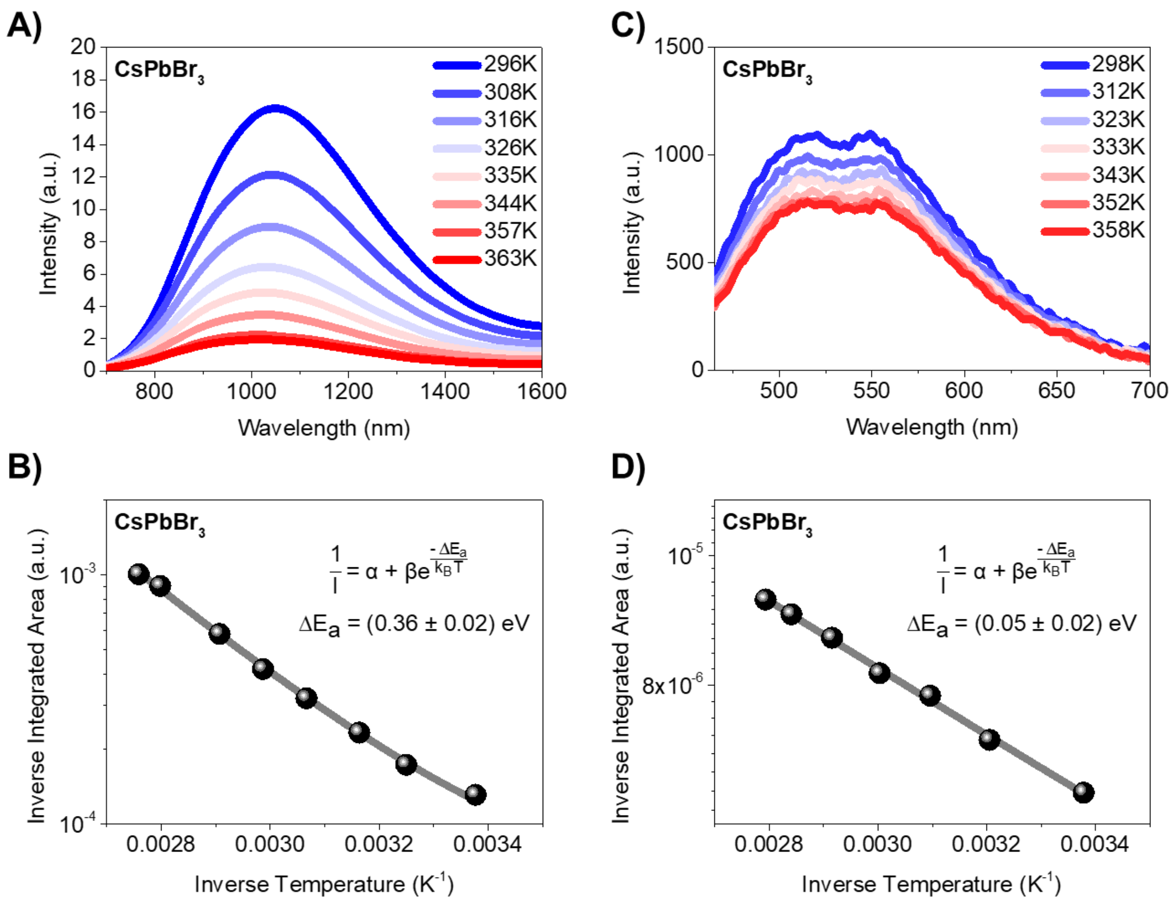

Figure S8. Temperature dependence of the VIS and NIR emission of $CsPbBr_3$. A) Temperature dependent NIR emission spectra of $CsPbBr_3$ (exc. 450 nm); B) Arrhenius plots of inverse integrated NIR emission intensity versus inverse temperature; C) Temperature dependent VIS emission spectra of $CsPbBr_3$ (exc. 450 nm); D) Arrhenius plots of inverse integrated VIS emission intensity versus inverse temperature.

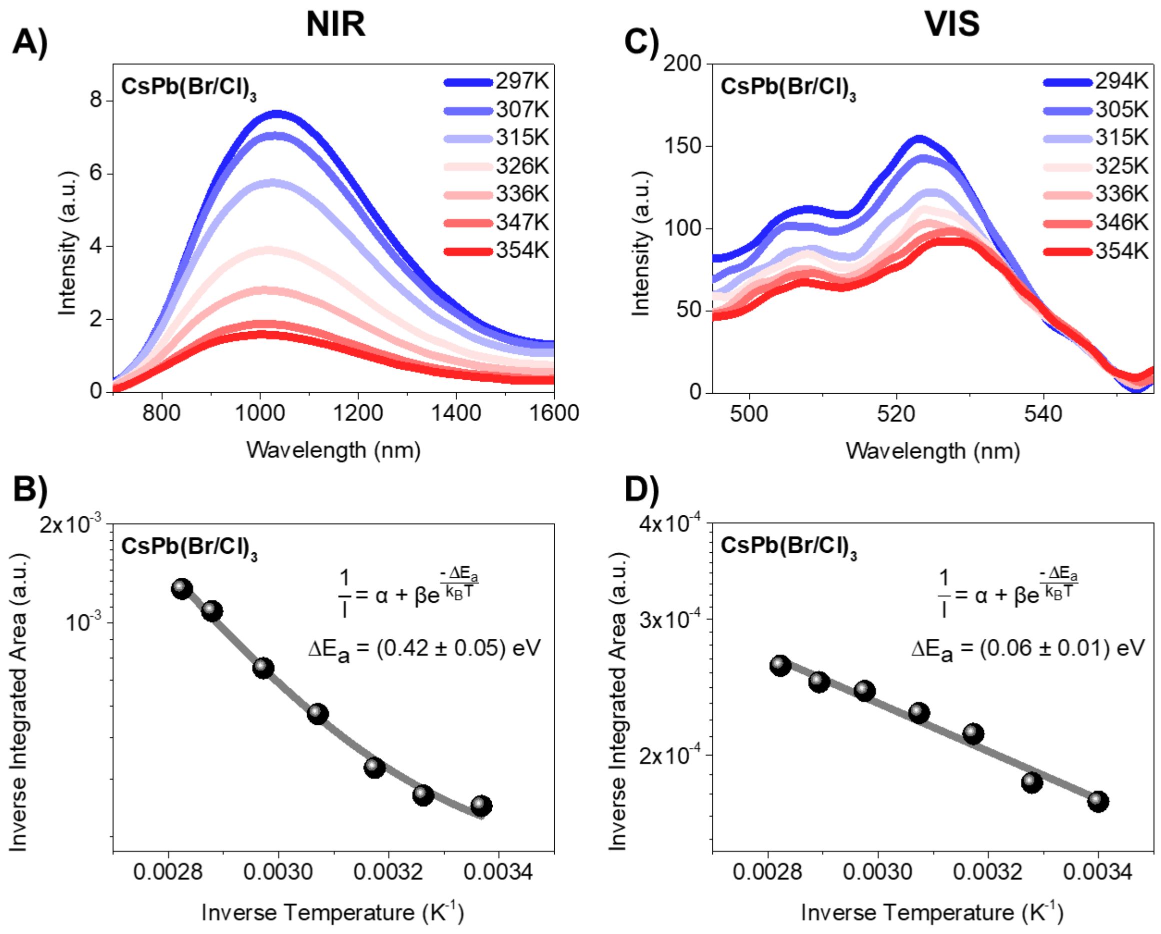

Figure S9. Temperature dependence of the VIS and NIR emission of $CsPb(Br/Cl)_3$. A) Temperature dependent NIR emission spectra of $CsPb(Br/Cl)_3$ (exc. 450 nm); B) Arrhenius plots of inverse integrated NIR emission intensity versus inverse temperature; C) Temperature dependent VIS emission spectra of $CsPb(Br/Cl)_3$ (exc. 450 nm); D) Arrhenius plots of inverse integrated VIS emission intensity versus inverse temperature.

Table S3. Parameters obtained from the analysis of temperature-dependent VIS and NIR emission spectra using equations (S4.6) and (S4.10)

**Sample**	**Detection wavelength (nm)**	$\boldsymbol{\phi_{em}}$ **at 298K (fixed)**	$\boldsymbol{\alpha'}$	$\boldsymbol{\beta'}$	$\boldsymbol{\Delta E_a}$ **(eV)**	$\boldsymbol{\frac{k_{NR}^{tot}}{k_R}}$
$CsPbBr_3$	465-700 (VIS)	$5x10^{-5}$	1900	$1.5x10^{5}$	0.05	$2x10^{4}$
	700-1600 (NIR)	$3x10^{-3}$	130	$2x10^{8}$	0.36	$3x10^{2}$

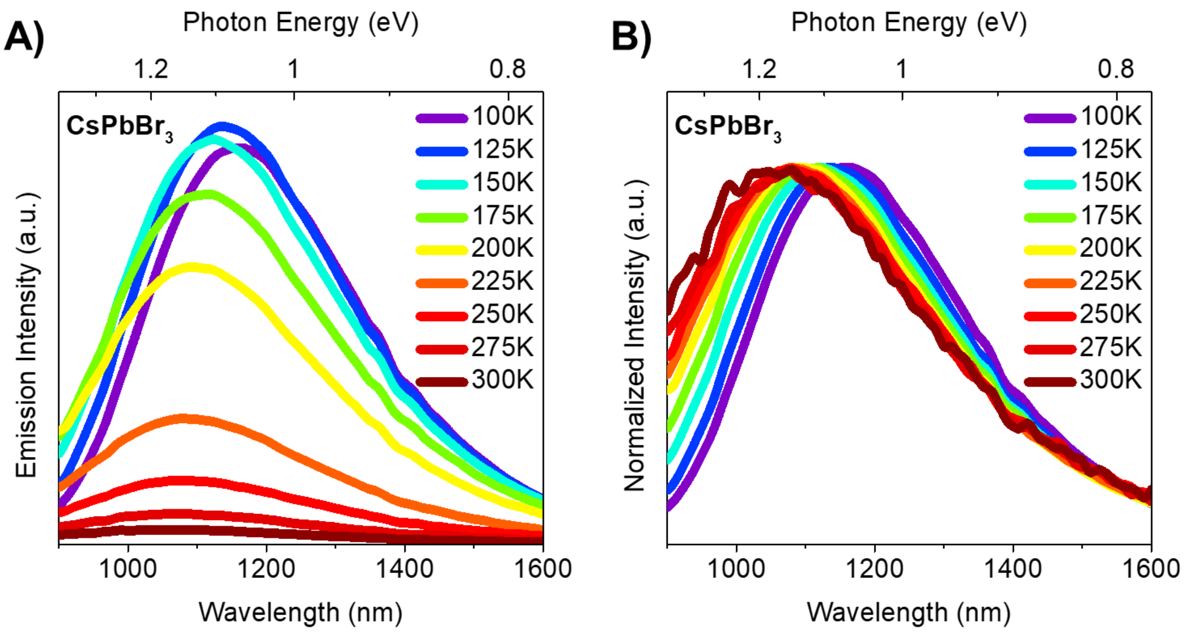

Figure S10. Temperature dependence of the NIR emission of $CsPbBr_3$ below room temperature. A) Temperature dependent NIR emission spectra of $CsPbBr_3$ (exc. 400 nm); B) Normalized temperature dependent NIR emission spectra of $CsPbBr_3$ (exc. 400 nm)

Upon decreasing the temperature from 300 K to 100 K, the NIR emission intensity increases significantly (approximately with a factor 27), due to the suppression of nonradiative relaxation at low temperature (Figure S10). Moreover, with decreasing temperature, the emission maximum systematically shifts from ca. 1070 nm (1.16 eV) at 300 K to ca. 1160 nm (1.07 eV) at 100 K. Increasing the temperature back to room temperature restores the original intensity and position of the spectrum, indicating that the change induced by the cooling is reversible. For sub-bandgap emission originating from a donor-acceptor pair transition between defect levels, a similar redshift with decreasing temperature has been reported in semiconductors, e.g. in ZnO particles.[22] Probably, there is a slight distribution in energies of the deep trap levels, depending on the local environment of the defects, which also accounts for the relatively large width of the NIR emission band. Upon cooling, as show by the increase of NIR emission intensity, nonradiative decay is suppressed. It is

possible that for different local environments, this occurs to a different extent, hence leading to an apparent redshift with decreasing temperature.

Excitation Power Dependent Photoluminescence Spectra

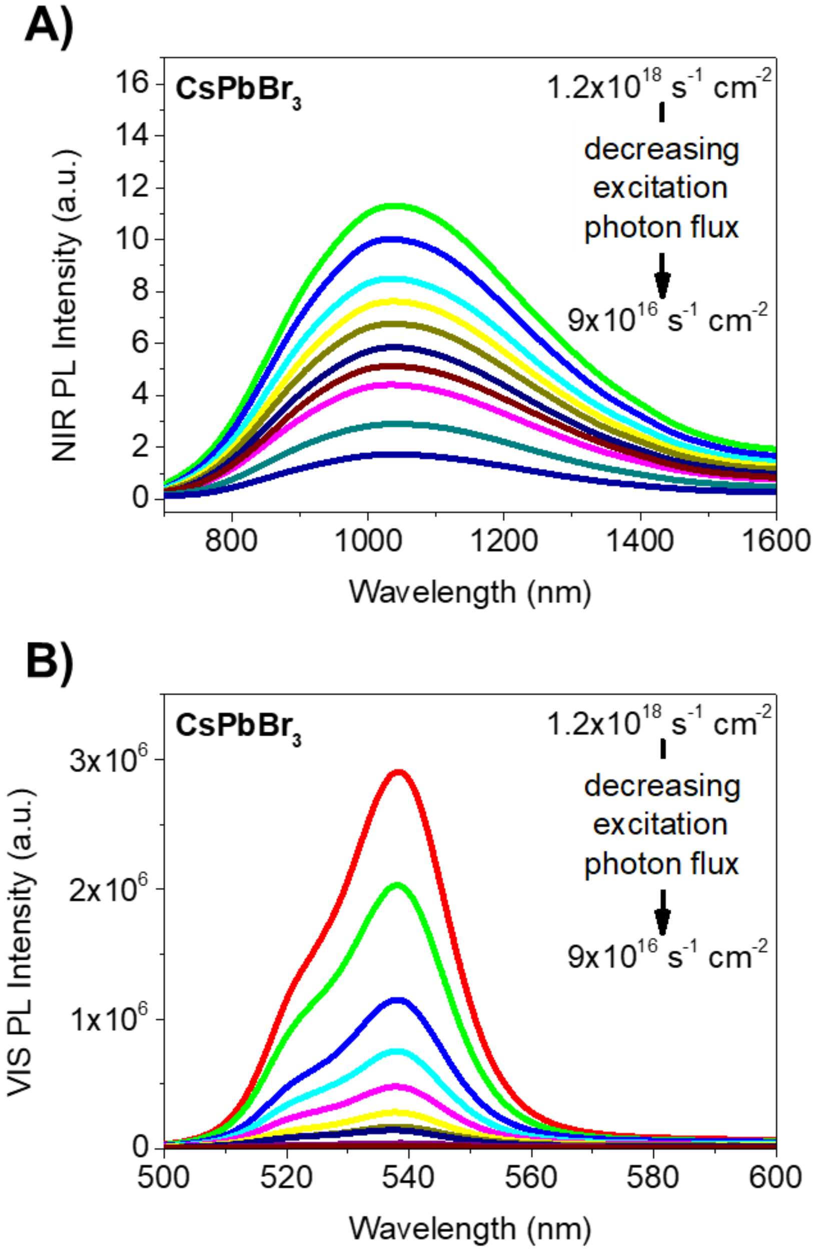

Figure S11. Excitation photon flux dependence of the VIS and NIR emission of $CsPbBr_3$. A) Power-dependent NIR emission spectra of $CsPbBr_3$ (exc. 375 nm); B) Power-dependent VIS emission spectra of $CsPbBr_3$ (exc. 375 nm)

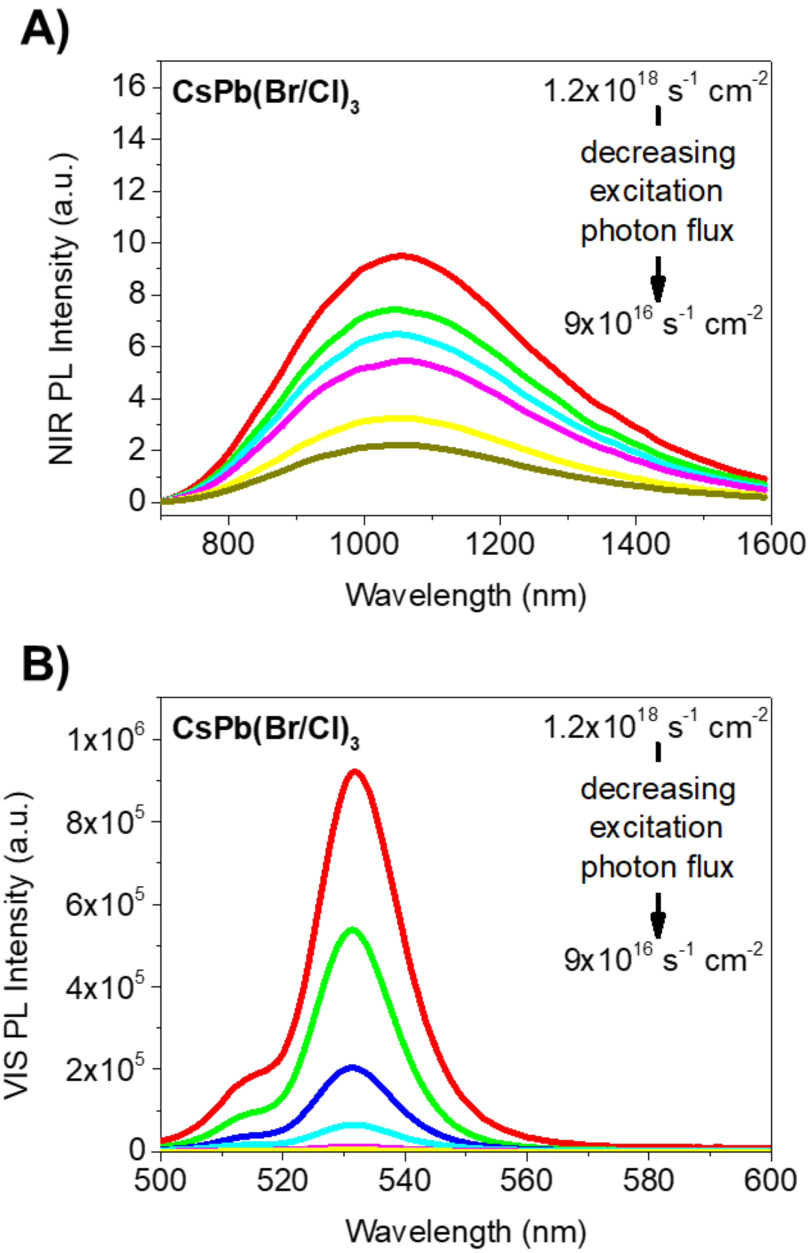

Figure S12. Excitation photon flux dependence of the VIS and NIR emission of $CsPb(Br/Cl)_3$. A) Power-dependent NIR emission spectra of $CsPb(Br/Cl)_3$ (exc. 375 nm); B) Power-dependent VIS emission spectra of $CsPb(Br/Cl)_3$ (exc. 375 nm)

Energy Diagram

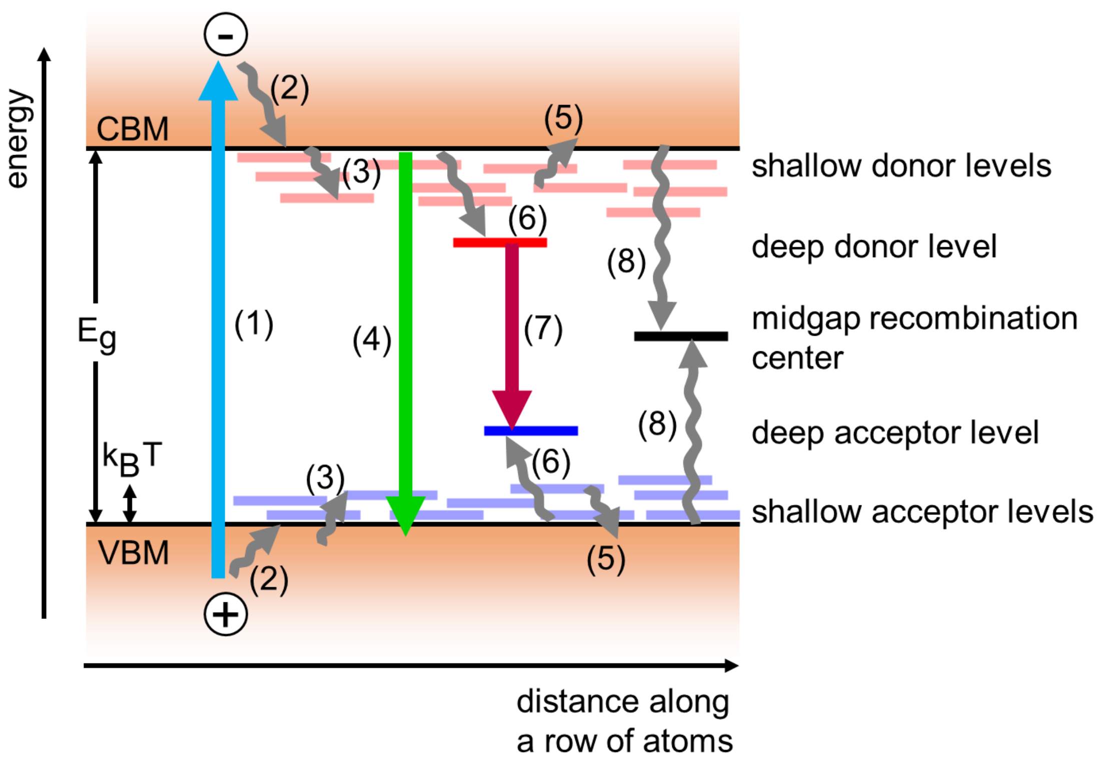

Figure S13. Schematic energy diagram depicting the photophysical processes after excitation in $CsPbBr_3$ microcrystals. (1) Above-bandgap excitation creating free e- (in CB) and h+ (in VB); (2) Thermalization to band edges; (3) Population of shallow traps; (4) Radiative relaxation (band-to-band or excitonic, VIS emission); (5) Depopulation of shallow traps; (6) Population of deep traps; (7) Radiative relaxation by donor-acceptor transition between deep traps (NIR emission); (8) Nonradiative relaxation through midgap recombination centers

References

(1) Huang, C. Y.; Wu, C. C.; Wu, C. L.; Lin, C. W. CsPbBr 3 Perovskite Powder, a Robust and Mass-Producible Single-Source Precursor: Synthesis, Characterization, and Optoelectronic Applications. *ACS Omega* **2019**, *4* (5), 8081–8086. https://doi.org/10.1021/acsomega.9b00385.

(2) Jia, Y.; Li, R.; Zhou, Y.; Zhao, S.; Yu, H.; Wang, J.; Lin, Z.; Su, H.; Zhao, N. Unveiling the Complex Evolution in Mixed Br–Cl Perovskite Precursors for High-Efficiency Deep-Blue Light-Emitting Diodes. *Small Struct.* **2023**, *4* (7), 2200393. https://doi.org/https://doi.org/10.1002/sstr.202200393.

(3) Yang, L.; Kruse, B. Revised Kubelka–Munk Theory: I. Theory and Application. *J. Opt. Soc. Am. A* **2004**, *21* (10), 1933–1941. https://doi.org/10.1364/josaa.21.001933.

(4) Klein, J.; Kampermann, L.; Mockenhaupt, B.; Behrens, M.; Strunk, J.; Bacher, G. Limitations of the Tauc Plot Method. *Adv. Funct. Mater.* **2023**, *33* (47), 1–19. https://doi.org/10.1002/adfm.202304523.

(5) Yang, Z.; Surrente, A.; Galkowski, K.; Miyata, A.; Portugall, O.; Sutton, R. J.; Haghighirad, A. A.; Snaith, H. J.; Maude, D. K.; Plochocka, P.; Nicholas, R. J. Impact of the Halide Cage on the Electronic Properties of Fully Inorganic Cesium Lead Halide Perovskites. *ACS Energy Lett.* **2017**, *2* (7), 1621–1627. https://doi.org/10.1021/acsenergylett.7b00416.

(6) Yakovlev, D. R.; Crooker, S. A.; Semina, M. A.; Rautert, J.; Mund, J.; Dirin, D. N.; Kovalenko, M. V; Bayer, M. Exciton–Polaritons in CsPbBr3 Crystals Revealed by Optical Reflectivity in High Magnetic Fields and Two-Photon Spectroscopy. *Phys. Status Solidi* **2024**, *18* (3), 2300407. https://doi.org/https://doi.org/10.1002/pssr.202300407.

(7) Jin, S.; Li, R.; Huang, H.; Jiang, N.; Lin, J.; Wang, S.; Zheng, Y.; Chen, X.; Chen, D. Compact Ultrabroadband Light-Emitting Diodes Based on Lanthanide-Doped Lead-Free Double Perovskites. *Light Sci. Appl.* **2022**, *11* (1), 52. https://doi.org/10.1038/s41377-022-00739-2.

(8) Würth, C.; Grabolle, M.; Pauli, J.; Spieles, M.; Resch-Genger, U. Relative and Absolute Determination of Fluorescence Quantum Yields of Transparent Samples. *Nat. Protoc.* **2013**, *8* (8), 1535–1550. https://doi.org/10.1038/nprot.2013.087.

(9) Fujimura, T.; Okada, K.; Nishiguchi, M.; Araki, Y.; Ikeue, T.; Sasai, R. Effect of Excited State Self-Quenching on Singlet Oxygen Photogeneration Using Nanosheet Surface Assembled Zinc Phthalocyanine. *Phys. Chem. Chem. Phys.* **2025**, *27* (8), 4328–4334. https://doi.org/10.1039/D4CP04126G.

(10) Nakagawa, T.; Okamoto, K.; Hanada, H.; Katoh, R. Probing with Randomly Interleaved Pulse Train Bridges the Gap between Ultrafast Pump-Probe and Nanosecond Flash Photolysis. *Opt. Lett.* **2016**, *41* (7), 1498. https://doi.org/10.1364/ol.41.001498.

(11) Mohamed, A.; Nishi, S.; Kawakami, K.; Shen, J. R.; Itoh, S.; Fukumura, H.; Shibata, Y. Exciton Quenching by Oxidized Chlorophyll Z across the Two Adjacent Monomers in a Photosystem II Core Dimer. *Photosynth. Res.* **2022**, *154* (3), 277–289. https://doi.org/10.1007/s11120-022-00948-1.

(12) Mohamed, A.; Nagao, R.; Noguchi, T.; Fukumura, H.; Shibata, Y. Structure-Based Modeling of Fluorescence Kinetics of Photosystem II: Relation between Its Dimeric Form and Photoregulation. *J. Phys. Chem. B* **2016**, *120* (3), 365–376. https://doi.org/10.1021/acs.jpcb.5b09103.

(13) Shibata, Y.; Nishi, S.; Kawakami, K.; Shen, J.-R.; Renger, T. Photosystem II Does Not Possess a Simple Excitation Energy Funnel: Time-Resolved Fluorescence Spectroscopy Meets Theory. *J. Am. Chem. Soc.* **2013**, *135* (18), 6903–6914. https://doi.org/10.1021/ja312586p.

(14) Braslavsky, S. E. Glossary of Terms Used in Photochemistry, 3rd Edition (IUPAC Recommendations 2006). *Pure Appl. Chem.* **2007**, *79* (3), 293–465. https://doi.org/doi:10.1351/pac200779030293.

(15) Lakowicz, J. R. *Principles of Fluorescence Spectroscopy*, 3rd ed.; Springer International Publishing: New York, 2006.

(16) Wang, W.; Su, J.; Zhang, L.; Lei, Y.; Wang, D.; Lu, D.; Bai, Y. Growth of Mixed-Halide Perovskite Single Crystals. *CrystEngComm* **2018**, *20* (12), 1635–1643. https://doi.org/10.1039/c7ce01691c.

(17) Su, Y.; Chen, X.; Ji, W.; Zeng, Q.; Ren, Z.; Su, Z.; Liu, L. Highly Controllable and Efficient Synthesis of Mixed-Halide CsPbX3 (X = Cl, Br, I) Perovskite QDs toward the Tunability of Entire Visible Light. *ACS Appl. Mater. Interfaces* **2017**, *9* (38), 33020–33028. https://doi.org/10.1021/acsami.7b10612.

(18) Baranowski, M.; Plochocka, P.; Su, R.; Legrand, L.; Barisien, T.; Bernardot, F.; Xiong, Q.; Testelin, C.; Chamarro, M. Exciton Binding Energy and Effective Mass of CsPbCl3: A Magneto-Optical Study. *Photonics Res.* **2020**, *8* (10), A50–A55. https://doi.org/10.1364/PRJ.401872.

(19) Stoumpos, C. C.; Malliakas, C. D.; Peters, J. A.; Liu, Z.; Sebastian, M.; Im, J.; Chasapis, T. C.; Wibowo, A. C.; Chung, D. Y.; Freeman, A. J.; Wessels, B. W.; Kanatzidis, M. G. Crystal Growth of the Perovskite Semiconductor CsPbBr3: A New Material for High-Energy Radiation Detection. *Cryst. Growth Des.* **2013**, *13* (7), 2722–2727. https://doi.org/10.1021/cg400645t.

(20) Clinckemalie, L.; Pradhan, B.; Vanden Brande, R.; Zhang, H.; Vandenwijngaerden, J.; Saha, R. A.; Romolini, G.; Sun, L.; Vandenbroucke, D.; Bonn, M.; Wang, H.; Debroye, E. Phase-Engineering Compact and Flexible CsPbBr3 Microcrystal Films for Robust X-Ray Detection. *J. Mater. Chem. C* **2024**, *12* (2), 655–663. https://doi.org/10.1039/D3TC01903A.

(21) Zhang, H.; Liu, X.; Dong, J.; Yu, H.; Zhou, C.; Zhang, B.; Xu, Y.; Jie, W. Centimeter-Sized Inorganic Lead Halide Perovskite CsPbBr3 Crystals Grown by an Improved Solution Method. *Cryst. Growth Des.* **2017**, *17* (12), 6426–6431. https://doi.org/10.1021/acs.cgd.7b01086.

(22) Mahesh, A.; Kumar, G. P.; Jawahar, I. N.; Biju, V. Temperature Dependent Photoluminescence Spectra of Nanocrystalline Zinc Oxide: Effect of Processing Condition on the Excitonic and Defect Mediated Emissions. *Chem. Phys. Impact* **2024**, *8*, 100456. https://doi.org/https://doi.org/10.1016/j.chphi.2023.100456.
